# What Makes a Partner Ideal, and for Whom? Compatibility Tests, Filter Tests, and the Mating Stability Matrix

**DOI:** 10.3390/bs10020048

**Published:** 2020-02-02

**Authors:** Lorenza Lucchi Basili, Pier Luigi Sacco

**Affiliations:** 1 Independent Researcher, 20 Chestnut Street, Cambridge, MA 02139, USA; lorenza.lucchi.basili@gmail.com; 2 Department of Humanities, IULM University, 20143 Milan, Italy; 3 Computational Human Behavior (CHuB) Lab, Bruno Kessler Foundation, Trento; Berkman-Klein Center for Internet and Society, Harvard University, and metaLAB (at) Harvard, Cambridge, MA 02138, USA

**Keywords:** tie-up theory, mating, super-cooperation, opportunism, constriction

## Abstract

We introduce a typological characterization of possible human heterosexual couples in terms of the concordance-opposition of the orientations of their active and receptive areas as defined by the tie-up theory. We show that human mating incentives, as characterized by widely adopted approaches, such as Becker’s marriage market approach, only capture very specific instances of actual couples thus characterized. Our approach allows us to instead explore how super-cooperation among partners vs. convenience vs. constriction may be regarded as alternatives modes of couple formation and cohesion, leading to very different types of couples with different implications in terms of stability and resilience. Our results may have interesting implications for future experimental research and for individual and family counseling.

## 1. Introduction

The human romantic imaginary places a big emphasis on the fact that mating is guided by the search for an ‘ideal’ partner, that is, somebody who not only matches the expectations and desires of those who search but also provides a perfect complementary fit, to arrive at a full harmonization as to intrinsic standards, such as personality traits and attachment styles, as well as to extrinsic ones, such as physical appearance and economic and social status [1]. Tellingly, the literature emphasizes how the conformity of a partner to certain ideal standards seems to have a predictive value for the stability of the couple [2]. However, to what extent do the ideal standards as expressed by the subjects reflect the real characteristics of the partners they will end up matched with, and the reasons that will lead them to match? The literature shows quite clearly that there is a marked sexual dimorphism in the characteristics that male vs. female subjects respectively tend to highlight as especially relevant in the choice of the partner: Women’s reproductive capacity (youth, physical aspect) for men, and men’s acquisitive capacity (wealth, socio-economic status) for women [3]. 

The limitation of this kind of analyses is, however, that they explore human mating preferences by directly interrogating subjects about the desirability of abstract partner characteristics, rather than of specific, individual potential partners placed in a specific real context. This implies, in particular, that subjects will tend to emphasize those ideal characteristics that correspond to their own conscious evaluation criteria, which reflect both personal preferences and the interiorization of norms and social conventions at work in their socio-cultural context of reference. The choice of the partner is, however, also guided by sub-conscious motivations whose effect is not appreciated in an abstract experimental elicitation, but that nonetheless play an important role in the specific situation of an interaction with a real potential partner. Once this further, scarcely considered dimension is kept into account, a more complex picture than the one drawn out by most mainstream theoretical approaches to human mating emerges.

At a conscious level, individual preferences and the influence of the social context closely interact in the fixation of the most desirable characteristics of a potential partner. Individual desires also tend to reflect an interiorization of social prescriptions, whereas prescriptions themselves tend to evolve under the pressure of changes in general individual orientations [4]. As human mating is a topic of special importance for so many different dimensions of social order, the collective regulation of the formation of human couples tends to be very prescriptive in many socio-cultural contexts [5], at times to the extent of leaving little or even no space to individual discretion [6]. In other contexts, and certainly in the contemporary secularized cultures that are typical of market democracies, much emphasis is placed instead on the freedom of partner choice, and on the necessity that people are put in the condition to mate with the partner they deem most congenial, beyond any possible social constraint [7]. Yet, even in societies where freedom of choice is loudly trumpeted, there exists many forms of conditioning whose action is difficult to eschew, and that are generally aimed at socially penalizing excessive differences between partners with respect to certain explicit or implicit ‘critical’ dimensions, such as, for instance, age, social and economic status, ethnicity, educational level, physical attractiveness, religion, and so on.

The conscious level tends however, as already remarked, to emphasize the objective desirability of certain individual traits, rather than to embed them within the dynamic framework of a specific relationship, where the combination of the individual characteristics of the involved partners is highly personalized and essentially unique. The match between the partners’ ideal models is not an objective, fixed feature, ruled by the presence/absence of a specific desirable trait, but is rather a process where each trait is considered and assessed within the overall bundle of partner characteristics [8]. A process-based vision of the ideal match between partners may be rather conceptualized as a (hard to achieve for real couples) shared pathway of mutual harmonization of psychological characteristics [9]. One should moreover observe that those ‘abstract’ individual and social standards of desirability are themselves far from immutable, and evolve at a speed that may widely differ across socio-cultural contexts [10]. The very balance between (dynamic, processual) partner compatibility and the desirability of the other’s personal characteristics on the basis of abstract individual and social standards is struck differently in different socio-cultural environments [11]. These highly specific contextual aspects of the human mating process may be appreciated with some difficulty by the theoretical approaches that focus their attention on abstract desirable characteristics, disconnected from concrete relational settings. 

At the sub-conscious level, the interaction between potential partners in the early phases of the process of possible couple formation leads them to test their reciprocal compatibility on the basis of criteria that do not correspond to those consciously stated, but that nevertheless have a precise adaptive value. The success of such compatibility tests does not depend on how closely the partner fits certain abstract criteria but rather on the evaluation of the global picture drawn through a number of signals that the assessing subject perceives about the other, in the light of one’s own psycho-biological characteristics. According to the tie-up theory [12,13], which provides the conceptual background of our analysis, for men, the relevant signals as to the compatibility of a given woman pertain to her personality traits, whereas for women the relevant signals as to the compatibility of a given man pertain to his physical characteristics and, indirectly, his genetic endowment. Both men and women, in suitable conditions, activate at the sub-conscious level specific compatibility tests on the potential partner, which, if successfully passed, pave the way to a possible mating-oriented interaction.

This does not imply, of course, that the characteristics that concur to determine the desirability of a potential partner at the conscious level play no role in the mating process—quite the contrary. The sub-conscious compatibility tests are accompanied by further tests, which can be conveniently named as filter or salience tests, whose purpose is exactly that of assessing the potential partner on the basis of the presence of certain desirable characteristics that play a focal role in the conscious preferences of the evaluating subject or in the social desirability norms that have been interiorized by the latter.

The compatibility and filter tests, respectively, reflect mutually independent evaluation criteria, which, however, may in some cases refer to a common informational basis (for instance, certain features of the potential partner’s physical aspect or personality traits). The filter tests generate many different evaluations on different dimensions, which are perceived as distinct from one another and later comparatively pondered through conscious reasoning. They may receive different relative weights, are not sexually dimorphic, and need not be all successful to ensure an overall positive evaluation of the conformity of the potential partner—and especially if the filter test, which is deemed the most relevant by the evaluator, provides a positive outcome. The compatibility test, on the contrary, is strongly sexually dimorphic and, although evaluating a number of different characteristics, aggregates its result into a single judgment of compatibility, which is not the product of a process of rational deliberation. Such a result may be reached, and may affect the evaluating subject’s behaviors, even before the latter becomes aware of it. The fact that the compatibility test operates at a sub-conscious level seems to be meant to ensure its non-manipulability by the conscious preferences and the social influences that are instead at the root of the filter tests. This guarantees the possibility that a potential partner, whose personal characteristics may have a high adaptive value in the economy of the couple, is being considered even if s/he does not conform to certain pre-defined individual or social expectations. On the other hand, the action of the filter tests may substantially influence the possibility to carry out the sub-conscious compatibility test — for instance, by precluding to those who fail it further opportunities of social interaction and close contact with the potential partner, which are indispensable for the operation of the compatibility test. Whereas the compatibility tests have a sub-conscious non-intentional nature, and are automatically activated in suitable circumstances, therefore not being subject to social or cultural influence, the filter tests are on the contrary determined by the socio-cultural environment, which will assign a higher salience at the conscious level to certain characteristics rather than to others. The content and nature of the filter tests will therefore be subject to a substantial cultural variability across time and space.

The possible conflict between the outcomes of the two types of tests discloses scenarios of particular interest, which, not incidentally, have been the object of considerable social attention for centuries. For instance, the analysis of the clash between the success of the compatibility tests and the failure of filter tests in the mating process, and especially of the consequences of such clash, is the topic of some literary masterpieces, such as Shakespeare’s *Romeo and Juliet*, where we find an iconic representation of the dramatic friction between the perfect poetical affinity of the two young lovers and the fierce opposition of a social context shaped by tribal hatred. A situation where the strength of the compatibility of the partners is such to overcome the obstacles posed by the most prohibitive filter tests, but whose fatal eventual outcome cannot but be the tragic epilogue not only of the couple, but of the partners themselves. In their capacity to capture the most subtle nuances of this kind of conflicts, certain romantic narratives like *Romeo and Juliet* acquire with time a strong social relevance, and tend to be transmitted from one generation to another as carriers of implicit social cognition value [14]. To the romantic narratives that obtain a certain level of social validation in terms of widely recognized salience is generally attached an experiential and learning value that may influence individual attitudes and expectations in real life mating processes [15]. 

The dynamic reciprocal adaptation between partners in the mating process, whatever its outcome, may open up very diverse interaction scenarios, which may entail very different levels of mutual cooperation in the construction of a couple bond. What distinguishes the most solid and resilient forms of human mating is a form of super-cooperation, where the partners are willingly locked into a stable cooperation mode [16] with altruistic components [17], which in the language of the tie-up theory will be defined as a double tie-up. The altruistic dimension that makes such super-cooperation more stable than other couple bonds is not innate but dynamically built through a highly adaptive process that we will call the tie-up cycle. One can find forms of cooperation between partners also in couples where no tie-up is normally observed, such as, for instance, in couples formed through combined marriage or in couples that are formed for convenience, e.g., to escape the control of the families of origin. However, such forms of cooperation do not present altruistic components and are more fragile with respect to external circumstances. The adaptive value of a super-cooperation between partners is instead that it offers a particularly stable, resilient environment for the joint rearing of the offspring. 

To understand in more detail how such a form of super-cooperation may emerge and what are the critical conditions that rule it, we observe that the cooperative dimension of the mating process contemplates a double layer: The one concerning the cooperation between partners, but also the one concerning the cooperation between the conscious and sub-conscious levels of each partner, with the concordance (or lack thereof) of the respective systems of incentives. It is possible that inner forms of conflict exist within one partner or both, so that, also as a consequence of a discordance in the outcomes of the respective tests, the orientations of the conscious and sub-conscious levels may pull in opposite directions. For instance, the conscious level of a female subject may appreciate a potential male partner for his wealth, while at the same time the sub-conscious test may signal a poor physical compatibility. An internal conflict may then arise in the subject, whose repercussions will inevitably be noticed also in the level of cooperation between partners and on the stability of the possible couple that might emerge from the interaction. To analyze the dynamic adaptation process between partners, it is then necessary to build a conceptual framework that allows an assessment of the possible types of couples that result from the combination of the level of cooperation established between the partners, and the internal levels of cooperation between the conscious and sub-conscious dimensions of each partner of the said couple. The super-cooperation that marks the tie-up calls for the simultaneous occurrence (concordance) of several cooperative conditions, and is thus quite restrictive. Such a framework may be summed up in a matrix, which will be called the mating stability matrix (MSM). The super-cooperation that comes with the tie-up can only be found in a few cells of the MSM, which enables us to understand how the structure of the couple is affected as the various cooperative conditions are gradually dropped.

It is important to stress that the dynamic adaptation of a couple does not necessarily consist in statically sitting in a single cell of the matrix, but it may also draw out a trajectory across some of the cells as a consequence of the evolution of the interaction due to its endogenous unfolding and to changes in external circumstances. The formation of a generic couple needs not be supported by super-cooperation, by mutual cooperation between partners, and not even necessarily by the unilateral cooperation of one of them. A couple may form in principle also in the absence of any cooperation from any partner, and even despite the failure of the compatibility tests, of the filter tests, and in the limit of both. Nonetheless, under certain conditions, cooperation might emerge in the course of the interaction, with varying levels of intensity and stability according to the circumstances. Our matrix is therefore to be seen as a sort of ‘state space’ that allows us to describe the extent to which the evolution of the interaction within the couple leads to more or less cooperative and to more or less stable outcomes, both from the point of view of the solidity of the couple and from the point of view of the internal coherence or conflict of each partner.

In this paper, we present a complete characterization in the matrix (MSM) form of the typology of possible couples that may be generated within the tie-up theory framework once we consider the articulation of all the possible configurations of cooperation/defection between partners and between the conscious and sub-conscious dimensions of each partner. This study then represents an extension of the tie-up theory that gains further insight into the relational dynamics of the partners within an already formed couple, and into their implications for the stability and resilience of such couples. These new analytical tools enable us to be more specific and operational in our criticism of the conceptualizations of desirable traits in mating processes that are based upon bundles of abstract and static characteristics, to consider instead a dynamic analysis centered upon the interaction of a specific couple in the context of its own social, economic, and cultural environment. With respect to the previous papers where the basics of the tie-up theory are outlined and discussed [12,13], the present paper therefore adds a new building block, which demonstrates the capacity of the tie-up theory to accommodate for the observed variety of heterosexual couples, and to appreciate their analogies and differences, blatant or subtle according to cases. The added value of this new theoretical development is its capacity to embed possible types of couples within a common conceptual framework that elucidates their relative positioning along a suitable spectrum. Such a positioning reflects into key couple functionalities vs. dysfunctionalities, and is determined by the complex combination of individual motives and social forces, showing how couples may be bound together by intrinsic drive or external constriction, and fueled by self-donation, opportunism, Machiavellianism, and so on. It also illustrates which kinds of couples are relatively closer to, or further away from each other, and in which sense. Across such a spectrum, we therefore find different degrees of couple stability and resilience, as well as of abuse and manipulation. Our matrix representation allows us to characterize the structural differences between any given kind of couple vis-à-vis any alternative one. The MSM matrix may therefore be useful to inspire further theoretical developments, to design experimental and empirical research, and also to develop new approaches to individual and family counseling.

The structure of the remaining sections is the following. Section 2 presents a brief discussion of the literature that tends to approach the mating process as a result of opportunistic choices with an implicit adaptive value, and of the limits of such an approach. In Section 3, we analyze the relationship between the cooperation within the monogamic couple finalized to the joint rearing of the offspring, and the dimorphism of the mechanisms that get men and women to bond into a stable couple, as characterized by the tie-up theory. In Section 4, we present and briefly discuss some of the main categories of filter tests. In Section 5, we introduce the mating stability matrix and briefly present its characteristics and main implications. Section 6 provides a final discussion and concludes.

## 2. The Opportunistic Couple: An Adequate Reference Model for the Study of the Formation of the Monogamic Heterosexual Couple?

In its current formulation, the tie-up theory can only be applied to heterosexual couples. It is important to stress that such a theoretical focus should not be read in any way in terms of heteronormativity. The only reason behind this choice is that extending the theory to other couples in the LGBT spectrum is not immediate, as it calls for significant further conceptual development. We are aware that this is a limitation of the theory, and we consider its extension in this respect an especially relevant and necessary avenue for future research.

Among the many approaches to the formation and dynamics of long-term human heterosexual couples, one of the most controversial but also most considered is that of Gary Becker [18], which explains the creation (and possible dissolution) of couple bonds in terms of the working of specific market mechanisms, whose competitive forces lead individuals to choose partners with comparable mating value. As the mating value of a given individual does not only depend on innate traits and characteristics but also on acquired ones (such as, for instance, wealth, power, and fame), and can be influenced by unanticipated shocks, like invalidating incidents and illnesses, such as stroke and dementia, which lead to caregiver burden on spouses [19,20], the individually available assets to be deployed in the ‘marriage market’ may vary from time to time. A given couple bond that looks stable for a given constellation of individual assets might thus be subject to serious stress, insofar as the value of one of the assets it builds upon undergoes major changes in either direction, positioning the individual in a different market segment from the original one, with a possible consequent dissolution of the couple and the creation of new couples with different partners whose mating values are aligned to the new situation. It is worth remarking that our reference to Becker’s ‘marriage market’ terminology does not amount to an assumption that marriage plays any kind of necessary role in the formation of a couple, and more generally in human mating. No part of our theory rests on this kind of assumption or implies this.

As mating values are only partially observable due to the high number of actual characteristics that contribute to the benefit that a given couple bond ensures to each partner on the basis of their preferences, it becomes necessary to focus one’s own evaluation on the subset of characteristics that are more amenable to observation and assessment in light of personal desirability criteria. With time, the acquisition of more information deriving from direct interaction makes one’s assessment of the partner more thorough and reliable, but on the other hand some of the partner’s characteristics evolve in ways that could in turn influence the evaluation. A widespread commonsense warns, for instance, that the elements of novelty (and thus of curiosity and discovery) in the relationship become rarer, one’s physical aspect changes with aging, the memory of past conflicts fuels misunderstanding and resentment, and so on. 

However, in the perspective of a competitive marriage market, even characteristics that as a first approximation may be held as innate and not amenable to change, such as the physical aspect, may be improved to increase one’s personal assets or to contrast their depreciation, for instance, by means of regular workouts [21] or aesthetic surgery [22,23], so as to increase or at least preserve one’s mating value and curb the potential competition of the carriers of more desirable traits [24]. Likewise, the psycho-relational aspects that may threaten the stability of the couple, such as the affective deterioration of the relationship, may be tackled by means of couple therapy or through the shared enjoyment of rewarding experiences. The psychological script associated to such compensatory strategies is, however, the ‘limitation of the damage’—that is, a program of reparatory actions that re-enact as closely as possible the ideal situation, identified with the relationship in its nascent state, heavily charged with psychological and sexual excitement, sense of novelty, and expectation of future gratification [25]—rather than the common cooperative pursuit of a growing physical and affective intimacy rooted in shared life experiences [26].

According to this script, the essence of the threat is the couple’s very coming of age, that is, the burden of the time spent together and of the past experiences, that causes a decrease of the excitatory value of the stimuli and gratifications deriving from within-couple interactions rather than an amplification, due to the growing reciprocal lack of interest of the partners [27]. Not incidentally, the possible reparative actions on one’s own physical aspect or psychological wellbeing, if particularly effective in terms of mating value improvement, might motivate the individual not to strive for the consolidation of the current couple relationship, but, on the contrary, to aspire to new more valuable (e.g., younger, more attractive) potential partners than the present one. Ultimately, the basic aspiration is experiencing new situations that bring back the high stimulation levels of the nascent couple.

Interpreting couple bonds as the outcome of the working of a ‘marriage market’ amounts to the assumption that the partners’ mating builds on essentially opportunistic motives. The partners are thus willing to keep the couple alive insofar as, and as long as, they do not find an alternative that seems to secure a larger expected benefit net of all costs related to the dissolution of the incumbent bond [28], including those deriving from the possible existence of (and consequently from the after-separation agreements concerning) joint offspring. By the same token, from Becker’s perspective, couple bonds tend to dissolve insofar as the partners become aware that their mating choices were based on partially unreliable information as to the characteristics of the other. Therefore, once such characteristics become apparent through direct experience, one realizes that they end up being less desirable than originally expected. In any case, the nature of the couple relationship is explained in terms of the matching of certain characteristics on the basis of the given preferences of each partner, rather than in terms of a dynamic process of mutual adaptation. This implies, for instance, that if for a given individual, certain absolute standards of physical attractiveness are what counts, also due to the social recognition that derives from being matched to a particularly attractive partner [29], how much effort the other puts into maintaining his/her own good looks makes little difference. The eventual breakdown of the couple as the former quits to mate a younger more attractive new partner is only a matter of time [30], whatever the actual previous history of a given couple relationship.

If considered from a disciplinary viewpoint other than that of economics, this vision appears as strongly limitative in at least two respects. On the one hand, human ecology points out how couple bonds have a cooperative rationale, which confers to the monogamic couple an important adaptive advantage [31], despite the consequent phenomena of antagonism and anti-sociality that are always potentially linked to cooperation [32]. The two partners commit toward the constitution of a stable bond that favors the joint rearing of the offspring, the more so the more the environmental conditions appear as hostile and challenging [33]. It is even possible to turn upside down Becker’s logic by defining ‘biological markets’, where the partner’s mating value primarily depends upon his/her capacity to credibly commit to a cooperative couple relationship, to also being helpful in non-emergency conditions, and to display strategic intelligence in gift-giving behaviors [34]. On the other hand, personality psychology underlines how the couple bond entails a complex process of dynamic adaptation [35], typically mediated by the individual psycho-affective developmental trajectories of the partners [36], leading to a gradual convergence between the partners in terms of emotional responses and personality traits [37], which in especially stable and accomplished couples may lead to a proper ‘fusion’ [38], that is, to a systematic prevalence of couple viewpoints and judgments over individual ones. Intense romantic love may be characterized in fact as a ‘system of attraction’ associated to specific dopaminergic reward pathways, and finalized to the development of high levels of self-sacrifice and (super-) cooperation within the couple [39]. This implies in particular that, in stable couples characterized by a strong romantic bond [40], the selfish evaluation of the desirability of the characteristics of the other is superimposed, and often entirely substituted, by an attitude that assigns to the other a central role in the shaping of one’s own authentic self, and thus ultimately of the most profound aspirations that feed the couple relationship [41].

An approach such as Becker’s one may therefore result not only misleading in its oversimplification of the motives and behavioral dynamics that lead to the formation or dissolution of a couple, but also be potentially harmful insofar as it acquires a prescriptive character that invites individuals to internalize the logic of the marriage market as the reference that guides their own mating choices [42]. The main reason that makes super-cooperation and even fusion possible in a successful long-term couple is the complex process of individuation and reciprocal re-cognition that leads one to consider the other not as a bundle of more or less desirable characteristics that contribute to define his/her mating value, but rather as a unique subject, with irreplaceable characteristics that cannot be trivially benchmarked against those of others [43], and that are truly defined by the common life experience, and thus, for instance, by the shared heritage of affective moments, common memories, accomplished parenting, and mutual donation experiences that contributed to transform to some extent the other in ‘a part of myself’ [44,45]. The emphasis on the uniqueness of the partner’s characteristics is also widely found in socially validated inter-generationally transmitted narrative accounts of the formation of the romantic couple [46,47]. 

The economic approach to couple formation and dissolution, in its poor capacity to keep into account the psycho-dynamic effects of the couple relationship on the partners’ own orientations, therefore suffers from basic conceptual limits that have to be reckoned with [48]. Clearly, this approach may effectively describe the rationale behind the formation and the dissolution of certain specific couples, and it is even possible that in certain historical and cultural contexts it may capture the ongoing social trends well. However, it is equally clear that its ambition to stand as the reference model that explains the dynamics of human couple relationships lacks proper ground, in that it tends to focus its attention on a specific, not fully representative mode of functioning of the processes of biological and cultural evolution that rule the stability of human couples.

The formation of a human couple is typically subject to the influence of a large number of factors of the most disparate nature. In certain contexts, the couple is formed by virtue of choices made by third parties, whereas in others the partners are the ones who decide. Also, it may happen that the social characteristics of the potential partners play a primary role in establishing their compatibility, whereas in other cases the personal characteristics lead the dance. Moreover, the choice of the partner may be founded on a large diversified basis of evaluation of many possible partners, whereas elsewhere the whole society may be organized to prevent one of the partners or both from acquiring any experience whatsoever that might allow comparative evaluations. The search for a conformal partner—that is, one who corresponds to a number of criteria dictated by the social context—may proceed according to a synergetic or antagonistic logic with respect to the search for a compatible partner, a partner, that is, who spontaneously causes a strong emotional arousal and becomes the object of a very focused attention and interest.

The social context may give importance to certain characteristics, assigning to them a specific salience in the process of couple formation, but such characteristics will not necessarily be those that will allow to the couple to last. Especially, a couple’s duration will not depend upon a mechanistic correspondence of given immutable characteristics [49] but will be the result of a process that, however conditioned by the social forces at play, will depend on the partners’ capacity and will to pursue a dynamic cooperation path built on each other’s ability to provide the other with the needed gratifications for an ever increasing physical and psychological intimacy. Each couple strikes a balance of its own between the partners’ reciprocal compatibility, and the conformity to the social norms and conventions as well as to the partners’ own preferences. In the present paper, we will introduce a taxonomy that characterizes possible couples exactly in terms of compatibility/conformity dyads. Such a taxonomy will enable us to show that those couples whose formation and dissolution dynamics reflects the opportunistic marriage market logic are far from being the rule, as Becker’s approach could implicitly suggest, but rather represent one among many possibilities, and not necessarily the best one in adaptive terms.

## 3. Methodology I: The Dimorphism of Tie-Up Mechanisms as an Adaptive Solution to the Formation of the Monogamic Couple

### 3.1. The Monogamic Couple: A Meaningful Exception

As pointed out by [31], the monogamic couple is a strictly minoritarian form of organization of family structure in the generality of human societies. In human socio-cultural history, the polygamic family is way more common and has, in particular, accompanied the development of increasingly complex and populated societies, sometimes been characterized by significant inequality in the distribution of social and economic resources, and where consequently, the number of brides functions as a clear signal of status. The monogamic vs. polygamic family represent the two possible stable equilibria of a model of gene–culture coevolution for a vast range of selection regimes [50]. To the current state of knowledge, the appearance and diffusion of the monogamic family has initially occurred in the European socio-cultural context, with the crucial transitional push associated to the Greek-Roman culture, which offers a strong cultural legitimacy to the monogamic family while at the same time maintaining a substantial openness toward polygamic out-of-wedlock relations [51]. Monogamy presents an important adaptive valence in that it shifts the male interest from the construction and maintenance of a harem of female partners, with a consequent necessity of a multi-lateral parental role with strong strategic components on both the male and female sides, to a more focused parental investment in the offspring generated by the sole female partner, while also reducing male contentiousness deriving from the substantial preclusion of stable mating for a large number of male subjects [52]. The relative weight of the selective pressures deriving from the male competition for female partners and from the advantages of monogamic parental investment, respectively, depends however on the environmental conditions [53]. 

The passage from the polygamic to the monogamic family lays the ground for a growing autonomy and self-determination of women, and for the reduction of male abuse [54]. It also significantly closes the gender gaps related to the access to economic and social resources [55]. Compared to the polygamic family setting, the monogamic one moreover brings about a remarkable improvement of the wellbeing and mental health conditions of women [56], as well as better educational results for the children, together with a reduced attitude to conflict for those grown in monogamic families with respect to polygamic ones [57]. 

The monogamic family therefore entails a substantial re-orientation from an extensive reproductive strategy founded on the competition for the access to, and control of, the female partners, to an intensive reproductive strategy based upon within-couple cooperation. It is meaningful that sexual differences appear as especially pronounced in Western societies, where marriage outside the family of origin is mandatory, vis-à-vis societies where kin marriage is allowed. In the former type of society, we expect more expression from genes that govern sexually dimorphic traits [58], and this may explain how the development of the monogamic couple has co-evolved with dimorphic mechanisms of incentivization of within-couple human cooperation. A basic problem connected to the socio-cultural selection of the monogamic family is indeed the male orientation toward the search for sexual variety in short-term mating [59], which may seriously threaten the long-term stability of the monogamic relationship. The capitalization of the adaptive advantages from joint parental investment within the stable monogamic couple therefore calls for an effective regulation of the male propension to form stable long-term couples vs. the male desire for sexual variety in short-term relationships [60]. Ultimately, the stability of a long-term couple built on a mutually rewarding physical and psychological intimacy, and more specifically, the stability of the cooperative process that supports it, requires some balancing of the (mostly male) drive for sexual opportunism [61], and especially so for subjects with high mating value [62].

The tie-up theory [12,13] offers a conceptualization of the dimorphic structure of the incentives to the formation of the couple, which under certain conditions may lead to a stable long-term mating where each partner is able to generate satisfactory rewards for the other, insofar as both are willing to persist in a dynamic self-catalytic couple relationship called the tie-up cycle. When the conditions for the creation of a stable mutually rewarding couple bond are not met, the tie-up theory allows the spectrum of possible alternatives to be characterized and classified, from the formation of a couple built on the pursuit of instrumental benefits or kept together by the coercion of the incumbent social norms and conventions, to the failed formation of the couple because of social or sexual opportunism or of poor compatibility between partners, and so on. In this sense, the tie-up theory does not have normative implications that suggest that a certain outcome may or should be more desirable than others, but limits itself to an analysis of under what conditions each of the possible outcomes is likely to emerge.

### 3.2. The Tie-Up Theory

The tie-up theory [12,13] moves from a precise characterization of the dimorphism of the male vs. female incentives to the formation of a stable long-term couple. Specifically, the theory introduces a distinction between direct rewards (deriving from the active/mostly conscious sphere of individual mating choices), and indirect rewards (deriving from the receptive/initially sub-conscious sphere of mating choices). In men, the active sphere is associated to the sexual dimension, whereas the receptive one to the psycho-emotional dimension. In women, on the contrary, the active sphere relates to the psycho-emotional dimension, and the receptive one to the sexual dimension.

The necessary but not sufficient condition that leads to a subject’s stable involvement within a long-term couple relationship is linked to the state of the receptive sphere, whereas the active sphere may play an important role in favoring, obstructing, or impeding involvement but cannot cause involvement alone. In particular, male involvement requires that the potential female partner successfully passes a test of psychological compatibility taking place in the male receptive area (M-RA). Analogously, female involvement requires that the potential male partner successfully passes a test of biological compatibility taking place in the female receptive area (F-RA). The success of the respective compatibility tests is the precondition for the emergence of the so-called tie-up (TU): A bond to a partner who is perceived as unique and irreplaceable, in his/her capacity to generate exclusive (indirect) rewards that substantially differ from those associated to any other kind of mating relationship. Specifically, in the presence of a potential partner that might meet the compatibility requirements, the RA ‘switches on’, starting to generate an indirect reward and activating the compatibility test. Such a switch-on occurs at a sub-conscious level, and therefore the subject may remain unaware of it even for a relatively long time. If the test generates information that is positively evaluated by the RA, this reflects into an increased production of indirect reward. Once a critical reward threshold is crossed, the compatibility test is successfully passed, the RA becomes excited and focuses upon the potential partner. If the compatibility test fails, the RA folds back to the quiescent state and does not generate indirect reward. Successfully passing the compatibility test makes the potential partner a viable candidate for a possible tie-up, whose formation depends on a further, intense flow of indirect reward through the interaction.

The passage from the successful test to the emergence of the tie-up presumes that the subject carrying out the test is not already bonded to someone else by means of a solid operating tie-up. In the latter case, the subject will, however, sub-consciously carry out the test when the situation is conducive to it, but this will not lead to a permanent excitement of the respective RA, if the RA is already excited for someone else. If instead there is no preexisting tie-up, or if such a tie-up turns out to be already weakened and fragile, the RA will be available for a new focused excitement toward the new subject who successfully passed the test. In younger subjects with little experience in the management of high peaks of indirect reward with their intense hormonal drive, the success of the compatibility test may very quickly translate into a tie-up without the need of further positive stimulation. When the peak of excitement of the RA is particularly high and the situation allows it, the success of the test may directly lead to the tie-up also in adult subjects.

This particular structure builds on a clear evolutionary rationale. The female biological compatibility test works toward the screening of the possible male partners from the viewpoint of the compatibility of their genetic endowment with the female one [63], and is carried out through the elaboration of a vast array of diverse signals, from physical aspect to posture, movement, voice pitch, bodily energy, expressive intensity, and haptic feel, with a special sensitivity toward those signals that transmit chemical information, such as smell and taste [64,65], or toward somatic traits associated with masculinity [66]. It is important to emphasize how the compatibility does not refer to characters that conform to a social standard of desirability—it rather prompts a subjective, unexpected, uncalculated response. A woman could then experience a high compatibility with respect to bodily traits that other women do not find attractive at all. This kind of test does not screen the potential male partner’s actual intention to form a stable couple, and thus exposes the woman to the risk of mating with sexually compatible but opportunistic partners. There is in fact a noticeable relationship between opportunistic personality traits in men and success in short-term mating [67]. 

The male psychological compatibility test instead works toward the screening of possible female partners from the point of view of personality traits, and of their possible psychological conformity with those of the male subject. Here, too, the test contemplates the elaboration of a number of signals of a varying nature, with opposite implications in different subjects. Male subjects could be turned on by psychological similarity or on the contrary by extreme diversity, by the capacity to support and be complicit or by challenge and competition, by spontaneity vs. self-control, by tenderness vs. transgression, by emotional intensity vs. sense of irony, by fragility vs. moral strength, or by camaraderie vs. sophistication, and so on. In a recent thread on Reddit (u/omg1223) where more than 10,000 men told about the key moment in which they understood they wanted to mate with their current partner, in all the answers rated as most meaningful by users, the described situation invariably focused upon the psychological qualities of the woman, and on their conformity to those of the man telling the story, rather than on her physical appearance. Tellingly, whereas in the case of short-term female partners, men value their attractiveness by considering the full range of bodily characteristics, for long-term partners, the attention concentrates upon facial characteristics, and thus on a bodily dimension more directly connected to personality traits [68]. Moreover, the (stated) personality characteristics of females directly influence male assessment of their physical attractiveness [69]. In the evaluation of personality traits, affinity counts in principle more than pro-sociality: For men with psychopathic personality traits, for instance, evidence of a psychopathic female personality may favor the success of the psychological compatibility test [70].

The evolutionary rationale of this dimorphic configuration of incentives deriving from the functioning of the respective receptive areas is that for women, the assessment of biological compatibility is a necessary condition to motivate the choice of a specific sexual partner, a choice that will rule out access to the alternative genetic endowments of other possible partners. This choice, however, entails very different levels of risk between the woman, who will have to bear the child and rear her/him anyway, and the man, who can spread his genetic endowment across a potentially high number of different partners. The female tie-up may be regarded in this sense, in a context of sperm competition [71], as a potential guarantee for the man against the possibility of displacement of his own sperm. The choice of the male partner by the woman will then be justified if the genetic endowment of the former signals a high level of compatibility, that is, tends to be based on an implicit assessment of the potential reproductive success of the couple that would be formed. This asymmetry in the allocation of the risks and costs of childbearing, which are mostly placed on the shoulders of the woman, is at the root of the social relevance that is generally associated to the exclusive concession of sexual availability by women but not by men, and thus to the reduction of female sexuality to an object of social exchange [72], as well as to the existence of ‘sexual double standards’ (strongly contextualized from the socio-cultural viewpoint) as to the social evaluation of out-of-wedlock sexuality in men vs. women [73]. For men, the assessment of the psychological compatibility is a necessary condition to motivate the reduction of sexual variety and the preclusion of short-term relationships, to the benefit of a specific investment in joint child rearing. Such investment will be justified if giving up alternative sexual opportunities will be compensated by other kinds of benefits for the man, and in particular by a stable, relationally harmonious union with a female partner, which ensures improved chances of successful parenting, and consequently of successful transmission not only at the genetic but also at the cultural level [74]—that is, of the parents’ values and beliefs, of the historical memory of the family, of ideas and world views, or behavioral norms in a variety of domains, and so on [75,76].

The success of the compatibility tests and the emergence of the tie-ups is, however, not sufficient to ensure the formation of a stable couple. To this purpose, the interaction that is finalized to the formation of the couple will have to be structured so as to incentivize both potential partners to cooperate in the reciprocal consolidation of the involvement of the other in the creation of the couple bond, and thus in the acknowledgement of the other as a potential long-term partner. The formation of a short-term or long-term couple, or the failed formation of a couple of any type, essentially depends on the kind of dynamic interaction that is established between the partners. The fact that a subject defines (and perceives) her/himself as interested in a short-term vs. long-term relationship expresses, not unlike the abstract preference for a partner with certain physical or social characteristics, the orientation of his/her active area, which is conscious and sensitive to social conditioning. However, this does not rule out at all that the subject may be actually involved in relational dynamics with a potential partner that does not fully correspond to the abstractly stated desiderata, and that nevertheless may lead to the formation of a couple whose nature is different from the one the subject claimed to be interested in. In other words, we have to distinguish between the categorial nature of the preference claims concerning possible couple relationships and the characteristics of a potential partner, and the individual nature of the specific interaction that leads to the possible formation of the couple. The fact that a subject comes to experience an interaction as finalized to a short-term vs. long-term relationship, or as not finalized to any kind of specific relationship, is the outcome of the context created by the interaction itself.

**Figure 1:**
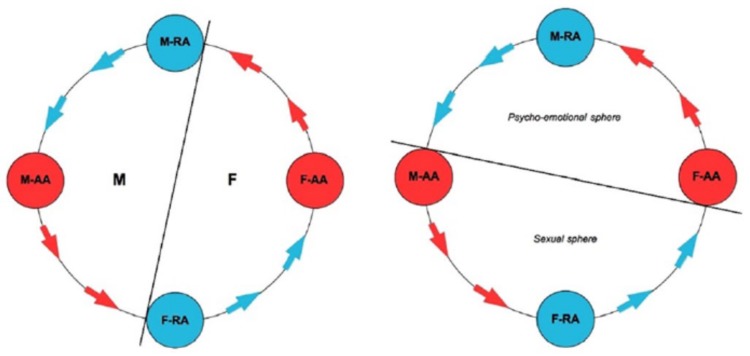
Representation of the tie-up cycle. Male vs. Female hemicycles, psycho-emotional vs. sexual spheres. M-AA = Male Active Area; M-RA = Male Receptive Area; F-AA = Female Active Area; F-RA = Female Receptive Area. Red arrows are direct rewards, blue arrows are indirect rewards.

The dynamic process through which the couple bond builds on or not as the interaction among possible partners unfolds is called the tie-up cycle (TU-C). In its natural direction, the cycle moves counter-clockwise and contemplates a sequence of direct and indirect rewards for each partner, which, if provided in the correct order, make the process self-catalytic, and thus driven by self-reinforcement through the cooperation among the partners until the formation of a stable couple bond (Figure 1). However, it is possible that the unfolding of the interaction threatens or precludes the completion of the cycle, its iteration or the momentum of such iteration, or leads to a simulated path for one or both partners, up to the possibility that one of the partners steps out of the cycle altogether, breaking it down. These other possibilities may lead or not to the formation of a short-term or long-term couple, but without a self-catalytic drive. The couple bond will therefore not be self-reinforced by the within-couple interaction, and it will remain intrinsically fragile even if it is formed in some way. The fact that the partners get tied-up at some point of the process is a precondition for self-catalysis, but it is always possible that even tied-up partners fail for some reason to move along the cycle in the correct way. 

Let us see now why the tie-up cycle has a characteristic direction, and why moving along it is related to the tying-up of the two partners. As already mentioned, in men, the active area has a sexual orientation whereas in women it has a psycho-emotional orientation. The direct rewards associated to such areas then have a sexual nature for men, and a psychological one for women. In the TU-C sequence, the generation of indirect rewards (that is, those associated to the RAs) precedes, both for men and women, that of direct rewards. This reflects the disadvantage of the woman as to the risks related to the mating. In particular, as it is possible to step into the TU-C from whatever position (that is, from any of the four areas of the graph), this implies that men can choose to enter from their M-AA, thus leaving behind their M-RA that would expose them to the possibility of being tied-up. If they are just aiming for a sexual reward, they can now simply move along the cycle counter-clockwise toward the (sexually orientated) F-RA. For women, however, it is the RA that is characterized in sexual terms, and consequently, female sexuality is always connected to a possibility of being tied-up—and the fact that the sexual F-RA precedes the psycho-emotional F-AA along the cycle inevitably entails a psycho-emotional involvement of the woman, that is, of her F-AA that receives the signal of the excitement of the F-RA in sexual intercourse due to the deployment of the flow of rewards along the TU-C. One could regard the characteristic mating-related interaction pattern of the TU-C as an alternative conceptualization of the dynamic process leading to the possible formation of monogamic couples with respect to existing theories, such as the stimulus-value-role theory [77]. In the stimulus-value-role theory, the process of couple formation is not driven by sexually dimorphic incentives, which are a central feature of the tie-up theory, but follows a different, partially conflicting logic. Designing experimental tests to establish which theory better describes the dynamics of couple formation, and in particular discerning whether or not the incentives behind such dynamics are sexually dimorphic, will be an important line of future research. 

The evolutionary rationale of the tie-up, when it occurs, is exactly that of offering the appropriate incentives to the formation of a monogamic couple. If the counter-clockwise direction of the TU-C may favor male sexual opportunism, it also explains the self-catalytic character of the cycle itself when it is effectively traveled along and iterated, with the consequent formation of the tie-ups. The fact that for each subject one’s own RA precedes the own AA in the TU-C also allows that indirect rewards, when the cycle starts from the RAs rather than from the AAs, sum up to the direct ones of the AA (which can also be obtained in the absence of a TU), thus operating as an incentive for further investment in the couple relationship rather than as an incentive to sexual or psycho-relational investments outside the couple. The specific incentivizing value of indirect rewards is due to the fact that whereas direct rewards are typically erratic, and therefore call for a periodical revamping of their elements of interests and of their consequent valence, indirect rewards tend to instead be reinforced with the consolidation of the couple bond, and to compensate the slumps of the direct ones in slowdown phases, thus preserving the overall level of reward from the couple relationship. Several iterations of the cycle may be necessary before a TU is created (or both). The iteration of the cycle in the absence of TUs requires a sufficient reciprocal interest between the partners in continuing their exchange of rewards and in not considering other potential partners, according to a logic of ‘gift exchange’ [78] that generates growing levels of intimacy and reciprocal trust.

As already remarked, successfully passing the compatibility tests then allows RAs to generate indirect rewards that may sum up to the rewards generated by the AAs. For instance, despite the physical aspect of a potential partner having to always obtain a pass from the M-AA, the excitement of the man’s M-RA sparked by the psychological characteristics of the woman may in turn fuel the M-AA’s sexual interest [79], even toward women that would not match the abstract standards of desirability of that particular man, and this will happen all the more intensely and consequentially the more the TU-C will have been iterated with success. Moreover, the direct reward obtained by a partner ends up, within a well-functioning TU-C, feeding in turn the indirect reward of the other partner who, if responding with an excitement of her/his RA (thanks to the success of the compatibility test), which feeds in turn her/his AA, eventually stimulates the RA of the former, thus closing the circuit. Therefore, if each partner is able to successfully move along her/his hemicycle of the TU-C, a self-catalytic positive feedback circuit will be established, leading to the strengthening and stabilization of the couple bond. The functioning of the TU-C is therefore finalized, through a sequence of cycles of progressive building up of the excitement of the respective RAs, to the creation of the tie-up for the man (M-TU) and for the woman (F-TU), or to its consolidation when one or both partners enter the cycle already tied-up. 

In principle, both sexes may kick off a TU-C by approaching and relating to an opposite sex subject, and this, as already remarked, may happen starting from one’s own AA or RA. The area from which one steps in therefore makes a big difference. Starting from the AA means that the RA may remain unengaged. This may be a temporary situation, so that the RA may eventually switch on as the cycle is traveled along and the RA is finally reached, thus starting to test the potential partner, or may be final, not switching on because the compatibility test is failed or indefinitely postponed. Moving from the RA implies, instead, a preexisting interest of the receptive area, and thus that the compatibility test has at least started or has already been carried out with success, or even that the tie-up has already been created even before the launch of the TU-C (that is, of the direct interaction with the opposite sex subject that has attracted the interest of the RA). The tie-up may therefore occur both within or outside of the TU-C, with or without interaction or the involvement of the potential partner. It is possible to get tied-up on one’s own as a consequence of the inadvertent stimulation of one’s own RA by an unaware subject (outside the TU-C), or on the contrary as a consequence of the intentional operation of the other’s AA on one’s RA (within the TU-C). 

A successfully functioning TU-C may be traveled along as follows. If the male partner is tied-up, and thus is motivated by the psychological intimacy with the partner (indirect reward) to seek sexual satisfaction within the couple relationship (direct reward), the sexual contact with a genetically compatible partner will generate indirect reward for the female partner, stimulating her AA to increase intimacy and psychological exchange with the male partner (direct reward). The latter will in turn experience an increase of his indirect reward, leading to further iterations of the cycle, strengthening his TU, and also favoring the F-TU if has not already occurred, through the consequent progressive stimulation of the F-RA. Analogous remarks apply if we move from the viewpoint of a tied-up female partner, or from the viewpoint of a not yet tied-up male or female partner.

How can we exemplify instead a not fully traveled along TU-C? If in the moment of generation of the direct (sexual) reward the man is not tied-up because of a failed compatibility test (which causes the man’s direct reward not to be fueled by a preceding indirect reward), the sexual contact will generate indirect reward for the (compatible) woman, leading the latter to increase her focus toward, and desire for intimacy with, the male partner (direct reward), but this request will not be favorably received by the man’s non-compatible RA and will then prompt a refusal of this kind of attention, and therefore a denial of the female direct reward. This implies that the flow of rewards in the TU-C stops every time it reaches the M-RA, remaining confined within the sexual sphere. As this situation keeps on repeating, the woman will experiment a growing frustration, which could lead her to refuse the re-iteration of a uniquely sexual relationship without further perspectives, and a growing decrease of the reward of the non-tied-up man, who in the absence of the contribution of his own RA to the strengthening of his direct reward, experiences a progressive sense of boredom and the dying down of his excitement, feeling the urge to seek new sources of (sexual) direct reward from other partners. Whoever decides to defect first, the TU-C will be inevitably dismantled. 

Likewise, if in the moment of generation of her (psycho-emotional) direct reward the woman is not tied-up because of a failed compatibility test, the relational exchange will generate indirect reward for the man, who will consequently go for a complementary direct (that is, sexual) reward, which will, however, not be granted by the non-compatible F-RA of the woman, who could refuse the inappropriate moves of the man. In this case, the flow of rewards will break down whenever the cycle reaches the woman’s F-RA, staying confined within the psycho-emotional sphere. The re-iteration of this situation will also lead in this case to the cycle’s dissolution, because of the systematic frustration of the man’s sexual expectations, inducing the latter to quit the relationship, or because of the growing unease of the woman toward a relationship that, not being fed by her switched-off F-RA, entails a progressive deterioration of her direct rewards, as they are accompanied by an unpleasant, more or less explicit request for sexual attentions. 

The specific structure of the active and receptive areas in men and women, and in particular their dimorphism and positioning within the TU-C, therefore offer an adaptive solution to the problem of female–male cooperation related to the formation of the monogamic heterosexual couple. The process that leads to the building of the TUs through the interlocking of male and female direct and indirect rewards with their different nature can also be regarded as an incentivization of within-couple male–female super-cooperation—that is, an unconditional reciprocal commitment [80] to reward the other both in sexual and in psycho-emotional terms as part of the couple bond [81]. This form of super-cooperation, by shifting the partners’ focus from self-centered to other-centered goals and benefits, has multiple adaptive implications: Securing a favorable environment for joint effective parenting [82]; eliciting mutual trust [83] and self-sacrifice for the sake of the other [84]; improving the couple’s resilience [85]; favoring cultural transmission of pro-social traits [86]; and even promoting the human development of both partners [87]. 

However, as for all forms of cooperation, however sophisticated, the structure of the TU-C allows opportunistic subjects who are able to manipulate it to obtain short-term benefits, which also include the woman’s impregnation outside the stable monogamous couple. The selective capacity of the TU-C is linked to its iteration: Long-term stable couples will be able to iteratively travel along the TU-C for a long time, whereas short-term ones will only go through it for a limited number of iterations, or will even (dynamically) confine themselves to a portion of the cycle without completing a full iteration. In this way, the TU-C favors the emergence of stable monogamic couples without jeopardizing the possibility of impregnation in non-stable couples or outside a proper couple. In stable monogamic couples, the TU-C also allows another important form of resilience: That against all those situations that compromise for relatively long periods a partner’s capacity to reward the other, such as incidents or invalidating illnesses or psychological suffering. The dynamics of a well-functioning TU-C may also be temporarily supported by a single partner due to its own inertia, which leads a partner to reward the other even in the temporary absence of reciprocity, and may in the limit keep on operating even in the case of a critical situation that is common to both partners, such as the loss of a child.

The TU-C may then be evolutionarily characterized as an incentive compatible dynamic relational mechanism [88] for tied-up potential partners, that is, it will be traveled along and iterated in a sustainable way only by those partners who receive a constant flow of mutually reinforcing direct and indirect rewards. A non-tied-up subject may simulate the TU and persist in the TU-C to reap personal benefits (for instance, if the other partner is rich, powerful, or socially desirable), but will have to manage an escalating conflict with her/his own non-excited RA, which fails to provide any indirect reward, thus calling for an effort, which in the long term is not sustainable, to constantly simulate a gratification that is instead real for actually tied-up partners. Other non-tied-up subjects who do not get special personal benefits from the persistence in the TU-C will limit themselves to openly manifest their opportunistic attitudes, trying to exploit the tied-up partner on the relevant level for their own AA, or they will simply quit the cycle. It is, however, important to underline how, unlike the simplest asymmetric information models, the incentive compatibility of the TU-C does not consist here in the discrimination of given behavioral types (for instance, subjects oriented toward short-term vs. long-term mating). It rather consists in the discrimination of choices that are endogenous to the relationship. In other words, a subject who might not be interested in persisting in the TU-C with a certain partner (to whom for instance s/he is not tied-up) might instead find her/himself persisting in another TU-C with a different partner to whom s/he gets tied-up. Subjects cannot be categorically classified as short-term vs. long-term oriented, even when their AA expresses itself in these terms, but will always contextually define their orientation with respect to a specific potential partner facing them. 

Recent research in neuroscience is moreover beginning to lay some key ground for understanding more deeply how the formation of couple bonds may be regulated and reinforced by specific neuroendocrine systems [89]. The literature has in particular focused its attention on the role of the oxytocin-vasopressin system in reproductive behaviors [90], in the formation of the couple [91], and in parental investment [92]. For instance, oxytocin increases male reward linked to the sight of the female partner’s face [93] and is strongly active in the regulation of interactive reciprocity in the early phases of couple formation [94]. Additionally, for testosterone, an important role has been highlighted in the context of complex regulatory mechanisms, both in the search for partners [95] and in the dynamics of parental rearing, in interaction with oxytocin [96]. Another line of research highlights an altruistic dimension of the couple bond (that interestingly resonates with the other-centeredness of the double tie-up): Subjects who are asked to endure pain for the benefit of their romantic partner experience no reduction in pain intensity, but a significant reduction in pain unpleasantness together with positive thoughts and pleasant feelings. This effect is mediated by the ventromedial prefrontal cortex, which infuses an affective dimension into the pain experience, transforming its nature [97]. The picture is still very fragmentary and intricated, and would call for an analysis that, for length and complexity, would be far beyond the scope of this paper. One cannot, however, rule out that the dimorphisms that have been found in the functioning of active and receptive areas for men and women could also have a clear signature in terms of sex-related differences in the functioning of neuroendocrine regulatory mechanisms. It is in any case important to point out how the topic of the neuroendocrine correspondences between direct and indirect rewards, compatibility tests, and tie-ups represents an important area for the future development of the tie-up theory and for the design of possible clinical experimentation protocols.

### 3.3. The Nature of Direct Rewards and the Factors of Social Influence 

Active areas are sensitive to social influence factors and to the instances of instrumental rationality. These are also the areas where, being involved in the conscious part of the decisional process, it is possible for subjects to exert a systematic activity of planning and control. The elicitation of the desirable characteristics of a potential partner will necessarily stimulate the active areas, in that it concerns a process of rational deliberation. Inevitably, then, we will witness a marked sexual dimorphism in the singling out of preferential traits, which, as pointed out by extensive cross-cultural research, has men privileging the physical aspect of the partner, and women emphasizing social resources, such as wealth and status [98]. However, as already observed, this does not imply that such abstract considerations actually translate into consistent choice criteria once subjects find themselves in the position of choosing real partners [99]. This latter choice depends, as already discussed, on the interaction between the rewards from the active areas and from the receptive ones, and in the absence of a sufficient level of indirect reward, the stability of the couple is seriously at risk of being compromised.

These remarks seem to be supported by the relative prevalence of subcortical structures and processes with respect to neocortical ones in the determination of the choice of the partner [100]. Between the abstract desirability of a partner and the actual formation of a long-term couple bond there is therefore a wide gulf. The formation of a couple bond implies a complex chain of favorable situations and circumstances. Not only do the reciprocal compatibility tests have to be successfully passed, but it is equally necessary for the partners to effectively implement a stable repeated cycle of cooperation, along which each one manages to appropriately feed the other with the needed indirect rewards, and vice versa. It is therefore far from strange or unlikely that a subject ends up forming a couple bond with a partner whose characteristics are different (and in some cases, very much so) than the ones s/he claimed to prefer at an abstract level. In particular, provided that indirect rewards are operating at a sub-conscious level whereas the claims about partner desirability are expressed consciously and only conforming to the logic of direct rewards, a paradoxical and only apparently counter-intuitive situation emerges, according to which each sex expresses preferences for aspects that are not those really crucial according to the logic of the RAs and of indirect rewards. In the absence of a suitable interpretive framework, such indications may therefore be deeply misleading for the purposes of personal and family counseling.

On the other hand, couples may exist not only because of the intrinsic value of the relationship but also because of its instrumental value. The formation of couples is not indeed guided only by the goal of finding a partner with whom to build a profound intimacy and unity of intent, but also by a number of other purposes linked to power, financial security, freedom from the constraints of the family of origin, social prestige, and so on. The process of signaling and strategic interaction that is at the root of the formation of the couple is thus potentially subject to any possible sort of strategic manipulation, simulation, and deceit, both on the male and the female sides. Such manipulative capacities are strategically oriented to systematically avoid a deep involvement in the couple relationship [101], and may bring significant advantages in the extraction of short-term benefits from the mating process [102]. In particular, pulling the partner into a position of unilateral tie-up puts the manipulating subject in a condition of special strategic advantage. A unilateral tie-up indeed allows the exercise of forms of exploitation to which the other could not be able to oppose even if becoming aware of them, insofar as such a tie-up keeps on being active and the RA keeps on generating indirect rewards, even in adverse circumstances and in the absence of a well-functioning TU-C. This is true in particular for female subjects, whose indirect reward is not linked to the dispositional attitudes of the partner but to biological compatibility, and might even be reinforced by a past history of humiliation, psychological, or sexual abuse at an early or developmental age [103,104], with its negative implications on self-worth, possibly mediated by self-blame [105]. It may thus happen that even in seriously dysfunctional and even abusive situations, the tie-up of the exploited female subject may be strengthened insofar as such situations entail occasions of substantial physical contact. 

In addition to individual manipulations of the process of couple formation, there are also those of a social nature. The social and material advantages related to the formation, maintenance, and dissolution of the couple are all potentially relevant in determining the rewards generated by the active areas. In particular, they may function as ‘filter’ factors in determining which potential partners have to be considered as salient for the purpose of checking their compatibility with the receptive areas. In a society dominated by a strong class stratification, for instance, it will be natural that the potential partner is sought among those who belong to the same social group. This will not make it impossible to consider, and even to choose, a partner belonging to a different social group but will imply that the formation of the couple bond must overcome not only the already mentioned obstacles but also those connected to the hostile environmental pressures that stigmatize the acquaintances between partners coming from too diverse social backgrounds. In this case, therefore, the actual formation and stability of the couple will have to face strongly selective conditions and will be particularly exposed to the action of disruptive forces. There will thus be a potential tension between the drives of the receptive areas and of those components of the active areas that are more sensitive to social influence, sparking a possible internal conflict for each partner, whose solution may open up very different scenarios and possibilities according to cases.

The tests of compatibility carried out by the receptive areas are then accompanied by further tests carried out by the active areas, which will be called ‘filter tests’. Indeed, if the possibility of the tie-up is regulated by the switching on of the RAs and by the success of the compatibility tests, the active areas have the faculty of pointing the selective attention of each subject toward certain potential partners instead of others, on the basis of the capacity of the former to conform to a series of norms and social conventions of particular relevance in the socio-cultural context under examination. In contexts characterized by marked gender inequalities, for instance, a female personality with strong autonomy and/or significant cultural and intellective skills could send a negative signal from the point of view of the ‘filter tests’, in that such personality traits prefigure a high propensity to question male leadership, thus threatening its social validation [106], and contradict the social expectations according to which men would be endowed with a superior level of intelligence in a wide array of dimensions [107].

The fact that a potential partner fails to pass one or more filter tests damages the possible formation of a couple much less than what would be the case with a failed compatibility test. However, such a failure may nevertheless make the operation of the TU-C so prohibitive in a hostile social environment to compromise the stability of the couple even in the presence of a profound compatibility between the partners as evaluated on the basis of the responses of the respective RAs, as widely illustrated by so many romantic tragedies. It is on these bases that a possible dilemma between compatibility and conformity emerges. If a couple turns out to be highly compatible according to the respective tests but at the same time must face adverse social conditions relative to the reciprocal positioning of the partners with respect to a series of socially salient criteria of conformity, the ensuing conflict is open to all possible solutions, which provide the basis for a meaningful typological classification of possible couples. It is of course possible that conflicts with an opposite sign arise, that is, the formation of a couple essentially due to the action of criteria of social conformity, which however failed to pass the reciprocal compatibility tests. Also possible are situations where compatibility and conformity are not in conflict but pull in the same direction, either in a positive (two compatible partners who form a couple that conforms to social norms) or in a negative sense (two incompatible partners, whose couple does not conform to social norms but that nevertheless are bonded due to the action of external forces).

Understanding in more depth the effect of various possible categories of filter tests and the consequences of the interaction between compatibility and filter tests for the typology of possible couples that may emerge, be them tied-up or not, is the goal of the next sections.

## 4. Methodology II: The Filter Tests

### 4.1. Concordance and Opposition between Areas

Both the active and the receptive areas tend to form an evaluation of an opposite sex subject that has raised the interest of one or both areas. Such evaluations are elaborated on the basis of the results of the respective tests, whose indications may be concordant or divergent according to cases. The concordance between the areas of a same subject indicates the absence of internal conflict of any nature, with respect to an actual or potential partner. Like in the case of two subjects who, despite the differences in their viewpoints on a same topic, fully agree in their overall orientations, AA and RA are in a dialogue as to the results of the respective tests, and end up being concordant when the final outcome of their evaluations is positive or negative for both.

As we have seen, the tie-up theory, in its basic formulation, stresses the crucial importance, for the purpose of the TU, of the compatibility tests carried out by both RAs, male and female, highlighting their sex-related specificities: The test of biological compatibility for women, and test of psychological compatibility for men. As these tests are not consciously activated, it is possible to intervene on them, at a conscious level, only as regards the conditions for their operation, by favoring them or, on the contrary, by inhibiting them as much as possible. Many social behaviors in different epochs and cultures were targeted at limiting or encouraging the compatibility tests. For instance, this was the case in the separation of many activities, such as school attendance, work, entertainment, and so on, into sexually homogeneous groups, or to the opposite, by facilitating meetings between opposite sex subjects through, for instance, dancing parties and receptions. A particularly isolating outfit, such as the burqa, in use in some Muslim cultures, has the social rationale of blocking male glances, but as a matter of fact also implicitly blocks any possibility for the woman to activate her biological compatibility test, which is extremely sensitive to the slightest sensory input. Sharing the same environment and carrying out common activities ends up facilitating the male psychological compatibility tests much more than what is instead possible in those traditional societies that rely upon fixed stereotyped characterizations of sex roles. In such societies, the possibility and expressive richness of the interaction is limited, as such interaction is shaped by social conventions that discourage a deeper communication. The test operated by the RA is indispensable for the tie-up to the partner, but as already discussed is not enough, alone, to guarantee the emergence and persistence of the TU-C. Even if both partners are reciprocally tied-up, that is, if a double tie-up (D-TU) emerges—an indispensable condition for the stability of the TU-C—this is once more not sufficient for the good functioning of the TU-C. The difference is then determined by the positions taken by the male and female AAs, in terms of concordance vs. opposition, to the results of the compatibility tests carried out by the RAs.

We call the tests carried out by the AAs filter tests as the term ‘filter’ denotes a condition of relational access. The success of the filter test for an opposite sex subject activates an attention pointer toward that subject. In other words, the filter tests of the AA are tests of salience whose function is to indicate how relevant a certain subject may be in terms of the formation of a possible couple, so much to be worthy of attention, up to becoming the center of the other subject’s romantic interests. Salience then refers to a subject’s potential as a possible, even temporary, partner. Among the many possible filter tests, each subject focuses on a certain number of tests that are particularly relevant for him/her, and assigns to them weights that reflect their subjective relevance. Unlike compatibility tests, filter tests are not sexually dimorphic, but the subject’s sex may affect their relevance, as other personal characteristics do. Clearly, the greater the number of filter tests that are successfully passed, the greater the overall salience reached by the subject. However, it can also happen that just one test provides such a high relevance to balance or even cancel the failure of the remaining filter tests. For instance, a very wealthy subject may be considered as a possible partner, even if lacking other elements of salience, insofar as wealth represents a crucial aspect for the specific AA that carries out the test.

### 4.2. Varieties of Filter Tests

Each filter test applies to a specific potentially relevant aspect for the choice of the partner, be it of a psycho-biological or socio-economic or cultural nature. An exhaustive list of all possible filter tests should be ample enough to include all the various dimensions that may influence the relevance of a potential partner in each possible epoch or geographical context, with their socio-economic and cultural variability. We will limit ourselves to consider some of the most significant tests, also in light of the existing literature.

#### 4.2.1. Physical Aspect

One of the filter tests that, as a rule, gets more salience for men is that based on the physical aspect, or better on the mating value of the woman [108]. The partner’s physical aspect is in particular considered by men a non-negotiable necessity [109]. The importance of this dimension is not surprising, also in view of the fact that the M-AA has a sexual nature and feeds upon direct rewards that are strictly linked to the man’s masculine identity and to his reproductive capacity [110]. 

Additionally, for women, the filter test that assesses the man’s physical aspect, despite the F-AA not having a sexual nature, may be especially salient. This is because it is a filter test that, in the case of the woman, although sending signals to the F-AA, in fact works as a bridge toward the biological compatibility test, or better favors it by channeling female interest toward a subject carrying a desirable genetic endowment [111]. We could then see this test, in the case of women, as a sort of pre-test of compatibility. It is, however, worth mentioning that each F-AA judges according to different parameters with respect to those relevant for the F-RA. The key aspects for a F-AA may, for instance, reflect the fads of the time [112], the expectations related to the age or the environment where the woman lives [113], sometimes even in a surprising contrast with the actual biological programming that drives the choices of each F-RA. It may therefore happen, for instance, that once the F-RA comes into play as well, a woman can also start appreciating physical features that are not fully congruent with those highlighted by the F-AA [114].

Physical aspect may be further decomposed in its constituents, not only innate ones but also acquired, such as expressive style [115], in its gesture, behavior, and posture aspects [116], which may, for instance, transmit, on the basis of the prevailing cultural standards, socially salient features, such as class and sophistication, but also sobriety vs. weirdness, and even rudeness and arrogance. Possible markers, for instance, are the elegance of the clothing style and responsiveness to incumbent fashion norms, or to the contrary lack of interest for one’s self-image, or even lack of aesthetic good sense. Another component that falls into the physical aspect domain is sexual magnetism. Both for men and women, irrespectively of the ruling canons of beauty and attractiveness, there are physical and behavioral traits that may render a subject especially interesting from the sexual viewpoint [117]. Such traits are strongly subjective, even if they may be at least partly linked to non-observable biological parameters [118,119], or to bodily gestures and postures that may be intensely attractive for a large number of people [120]. Finally, there may be certain desirable physical traits, also in terms of dimension, color, and form, which may also draw social recognition once they are made the object of general appreciation [121]. Expressive style and sexual magnetism, in their evocation of certain personality traits, may also be seen from a male perspective as a pre-test of psychological compatibility.

#### 4.2.2. Wealth and Status

If physical aspect generally gets a higher salience for the M-AA, wealth and social status have a comparable weight and frequency in terms of salience for the F-AA [122]. Like the physical aspect for men, wealth and status is a non-negotiable aspect for women [109]. Clearly, wealth and status may be seen as desirable in themselves, but for women, due to their historically greater difficulty, in the various epochs and social contexts, to own the necessary resources not only for themselves but also for their future children, they become one of the most discriminating and crucial filter tests [123], especially when the partner represents the only opportunity to reach a stable desirable source of livelihood and social position [124]. The growing empowerment of women as to control of economic resources and financial independence in secularized societies may reduce the salience and relative weight of this kind of filter test for female mating choices [125]. 

Of course, for men as well there may be a similar motivation that leads to consideration of the advantage of mating to a rich/high social status female partner (a classical fictional example is Maupassant’s Bel-Ami [126]). Much depends on the starting conditions and on the perspectives of each single individual with reference to the context and environment one lives in. However, the filter test that assesses the partner in terms of the opportunity of access to material resources presents a relatively high discriminating relevance for the overall success of the AA-administered tests, and in particular one that may even reinforce a possible opposition of the AA to the orientations of the RA.

#### 4.2.3. Personality and Intelligence

Personality, intelligence, and cognitive skills cover a vast range of very important aspects of the filter tests, which include specific features of special relevance, such as emotional intelligence, charisma, sensitivity, value orientations, and experiences [127]. When there is not a need to invest all attention toward social status and wealth, the F-AAs are very focused on singling out a partner that successfully passes the filter tests for personality and intelligence [128], possibly concentrating upon traits that are especially desirable in the social context of reference [129]. Often, women, when asked about their ideal partner, typically start from personality traits such as kindness, affect, acumen, honesty, psychological balance, agreeableness, sense of humor etc. [130], and seem to lend less explicit attention to aspects such as sexual attraction, which is instead the basic criterion of the biological compatibility test carried out by the F-RA [131]. This depends on the fact that the F-AA is active in the psycho-emotional sphere of the TU-C, and then expresses itself in conformity with its sphere of reference, in addition to the non-negligible fact that the passiveness of the RA generally causes it to retract and to be unwilling to be exposed through its expression, especially if it finds itself in a switched-off not-excited phase. Likewise, the M-AA, being instead active in the sexual sphere of the TU-C, to the same request for a description of the ideal partner instinctively focuses primarily on physical aspects rather than on personality and intelligence [132]. However, only the latter will in fact be evaluated by the M-RA’s test of psychological compatibility. The statement of principle, in practice, often does not correspond to the most satisfactory choice that will actually be made, not because it is not possible to find an intelligent man with a good personality who is also sexually attractive for a given woman, or a sexually attractive woman who is also intelligent and capable of psychologically stimulating a man, but because mere intelligence or good character do not suffice to determine the F-TU in the absence of sexual attraction. Or, to the contrary, mere sexual attraction, however intense, does not suffice to determine the M-TU in the absence of a psycho-emotional attraction in the man.

Consequently, men who choose their stable partner not keeping into account their compatibility with the personality traits and with the intelligence of the latter will give birth to a TU-C with feeble chances of persistence, whereas the woman who chooses her stable partner without taking into account her biophysical signals may be especially susceptible to spousal infidelity if she gets finally tied-up to another male subject.

#### 4.2.4. Social Network

The network of acquaintances and relational connections represents a social resource, albeit intangible, which may prove to be as beneficial as status and wealth [133]. Moreover, the filter test that assesses the extent to which a potential partner is embedded in a certain social network [134] and contributes to its cohesion through pro-social behaviors [135], also provides indirect information on her/his personality (for instance, on her/his sociability and extraversion), on present or future financial and professional opportunities, or even on the level of appreciation by opposite sex subjects. In the case of the woman, much depends on the socio-cultural context: If it is egalitarian, gender equality leads to rather similar criteria for men and women in terms of appreciation from having a vast relational network, even if a woman with an overextended network of male friends is susceptible of a less favorable consideration [136]. In contexts without gender equality, men tend to expect that the woman’s social network is essentially mediated by the family [137], and in the most restrictive conditions a network of relationships outside the family of origin becomes a negative factor in terms of salience, or even a serious social transgression [138], which may also lead to a global failure of the filter tests of the M-AA. As regards men, if they are embedded in a vast network of social connections, this is not seen negatively by women, and even if such a network includes a number of female acquaintances, this might even increase the female interest toward the man [136], even more so if the man already has, or had, an attractive female romantic partner [139]. The reasons behind this difference may be traced back to a propensity to competitiveness, which in the case of women generally leads them to adjudicate the attentions of a much sought-after man, to win sexual competition against other female rivals. In the case of men, when it is a matter of being emotionally linked to one woman only instead of seeking sexual variety, psychological competitiveness intervenes, which in the presence of too many male competitors entails a loss in the value of the uniqueness of the man’s own exclusive compatibility with the woman. 

Women appreciate men with a wide spectrum of compatibility both in sexual and psychological terms, as this is not only an index of a genetic endowment that is generally valued at the community’s level but is also a sign of a subject that carries highly rated salient (for the F-AA) psychological traits [140]. 

Competitiveness toward one’s own sex in this case is fueled by the fact that the F-AA has a psycho-emotional nature. In the case of men, as M-AA instead has a sexual nature, it is sexual competition that does not generate insecurity, ending up feeding the interest toward a woman that displays a high mating value so that many other men are also attracted—this is because the AAs generally get excited if they prevail in a competition. However, as regards the psycho-emotional sphere to which the social network pertains, men occupy it in a passive position (M-RA), and therefore they feel much less like competing in this regard, preferring situations of relational uniqueness and specificity that motivate their renounce to sexual variety. A woman showing an excessive propensity to the psychological compatibility both in a wide network of social connections and, especially, toward many possible male subjects, in addition to raising the suspicion of an overactive tendency in the sexual sphere, reduces the exclusivity value of the man’s psychological compatibility with that specific woman. In other words, for men, being bonded to a single woman is no longer a mere issue of sexual conquest, rewarding for the M-AA, but becomes a problem of individuation of a partner, who is also and primarily psychologically compatible, with a uniqueness value that cannot be shared with others [141]. In particular, as already remarked, this exceptionality must be pronounced enough to push men to give up to the value of sexual diversity so much appreciated by the M-AA. For women, a similar insecurity is related to the F-RA in the sexual sphere, and indirectly to the psycho-emotional sphere if the M-TU may be compromised by the psychological attraction toward another woman.

#### 4.2.5. Evidence of Tie-Up of the Potential Partner

Knowing that a potential partner has tied-up to a subject represents a direct reward for the subject’s AA, all the more so the more the tied-up subject proves to be salient and worthy of attention for the AA. Finding a tied-up partner is a guarantee of enhanced stability for the future couple, and the TU corresponds to a further quality of the partner, as it makes more remote the possibility of being exploited, but on the reverse, it also makes a possible exploitation of the tied-up partner easier. For this reason, courtship played, and with all due qualifications still plays, an essential role in successfully passing this kind of filter test for males and today increasingly also for females. Historically, courtship has to do with socially conspicuous rituals (such as singing serenades, or giving flower gifts), and more generally with social displays of generosity and altruism [142]. 

Courtship may in fact be seen as a sort of mise-en-scene of a presumed TU, as well as of the value of the courting subject. The ritual of prescribed courtship behaviors is designed to enact the actions and the focus of interest that are representative of a tied-up subject who is worthy of attention. Accepting the courtship of a man and reciprocating with signals, such as smiles, glances, and explicit emotion, which in turn emulate the tie-up, may be a way for a woman to display her own interest and to communicate to the man that he has successfully passed the filter tests. In this sense, courtship may be interpreted as a signaling game under incomplete information [143].

This filter test is, however, the most insidious, as it is easily falsifiable when someone wants to simulate her/his own TU for manipulative purposes [144]. Some rigor is therefore called for in testing, as it is symbolically well depicted in the popular culture of historical fairy tales, where the suitor that aimed at marrying the princess had to pass a number of very challenging trials—nothing else than filter tests in fact, which in addition to assessing qualities, such as intelligence and courage, were targeted at exposing the concealment of undesirable traits, such as greed or Machiavellianism; that is, traits that signaled a likely intent of exploitation of the partner in the absence of a D-TU. 

#### 4.2.6. Familiarity

The filter test on familiarity has the purpose of signaling the presence of heritable traits from the biological or behavioral viewpoint that might affect the future offspring. For this reason, familiarity often has a valence of a negative filter, ruling the exclusion of subjects who are carriers of genetic or behavioral defects that are directly observable in the family of origin (illnesses, bodily malformations, mental health problems, domestic violence, criminal attitudes, alcoholism, drug or gaming addiction, etc. [145]). Familiarity may, however, also assume a valence of a positive filter when a potential partner may be associated to heritable, directly observable, and especially valuable family qualities, such as intelligence or sociability, favoring the possibility of assortative mating [146]. Especially in societies where families, and parents in particular, play an important role in the choice of the partners of their siblings, the application of this type of filter may also be externally conditioned, determining an assessment of the whole family background of the subject under examination as a possible son- or daughter-in-law. In this kind of society, orphans tend to be disadvantaged as to filter tests like this due to the uncertainty concerning possible familiarity issues in the family of origin, about which there is a lack of reliable information.

In addition to heritability, familiarity may also entail a filter that blocks the access to representatives of hostile clans, in the case of long-term tribal hatred, rivalry, and hostility, that may carry over to the potential partners [147].

A different factor of familiarity may be the salience of potential partners whose traits reflect those of one’s own opposite sex parent, an effect that seems to have some relevance in the functioning of filter tests, and especially for subjects who have not experienced recurrent refusal from their opposite sex parent [148], even if the actual dimension of such an effect seems controversial [149].

#### 4.2.7. Social Conformity

The social conformity of a partner is evaluated in relation to social and cultural prescriptions. Especially in certain societies, the conformity to social rules is an almost inescapable condition so that the partner may be salient with respect to the AA’s filter test, as this is an indicator of the extent to which the potential partner is actually integrated and worthy of social respect [150]. The less likely the possibility of deepening the direct acquaintance with the future partner, the more potentially relevant the assessment of his/her personal capacity to conform to certain prescriptions becomes, also indirectly signaling the absence of conditions of psycho-social deviance. Moreover, there is the possibility that belonging to a certain family or social group may assume such an importance to constrain the filter test of social conformity to a collective rather than individual approval [151], thus coalescing into a social approval filter test (see below). It must be pointed out, however, that in societies characterized by less stringent norms where it is possible to assess the socio-psychological traits of a subject in the light of a much richer and more varied context of direct acquaintance, and in which there is a tendency to value creativity and originality, the lack of social conformity may instead become a point of interest as it denotes an interesting personality and uncommon, independent, and innovative intellective skills [152]. Still, the social conformity filter may end up disguising the actual personal characteristics of a subject, making it more difficult to assess a real psychological compatibility and, as a matter of fact, in certain societies may even become a substitute for it.

#### 4.2.8. Moral Responsibility

This filter test does not represent a possible obstacle to the formation of the couple but may cause its dissolution in that it is activated along the process, as one of the partners realizes that the union is damaging, for some reason, the opposite sex partner. The altruism toward the other partner, which is an outcome of the TU itself, may lead to the sacrifice of personal interest even in terms of a painful subtraction of one’s own TU from the TU-C, for a moral purpose and to the benefit of the other. It might in the limit even be responsible for extreme choices, such as certain forms of suicide [153]. Such a decision is made in loneliness by an AA that is concordant with its own RA and with the partner’s areas, but that for the superior interest of the latter decides to not accommodate the desires of her/his own RA and opts for an escape from the TU-C that often simulates a false condition, that is, a false absence of a tie-up.

#### 4.2.9. Social Approval

The social approval filter does not fall within the category of the tests carried out by the partners, but it is, however, able to deeply affect their AAs. The couple may in fact become the scapegoat for preexisting unresolved conflicts, at the family or social level. An example of an application of this filter is the interracial couple that is not accepted by parents and relatives because of persisting racial prejudices. More generally, the issue of social acceptance arises whenever the partners represent two different worlds from the cultural, financial, religious, ideological, and social viewpoint, with little possibility of fruitful intersection, sharing, and integration [154]. Within the social approval domain also fall the previously described tests of social conformity and familiarity, whenever they are carried out outside the couple and then coalesce with other factors of social approval.

### 4.3. Filter Tests and Rationalization of Mating Choices 

Our survey of some of the main filter tests, inevitably brief and with a mainly exemplificative value, points out how these are carried out at a level of awareness far bigger than for the RAs’ compatibility tests, which may surface to the conscious level even long after they have been concluded. The filter tests generate flows of thought that may be compared to proper forms of reasoning, with their justification and argumentation apparatuses. This is why they are often mistaken for the real motivations that should determine the formation of an ideal couple, only to be surprised by its rapid wear and tear, which will in turn be rationalized through ex-post, ad hoc justifications and arguments. The outcomes of the compatibility tests may instead condition the subjects’ choices in ways that can hardly be supported by rational argumentation, and may be mostly understood only through the effects they produce at the psycho-neuro-endocrinological level. This because the test carried out by the RA occurs at a level of conscience that lies below the threshold of immediate awareness, and as it does not require any reasoning, it then appears as irrational and lacking any apparent explanation, just like one often describes the process of falling in love.

The concordance between the areas of a same subject implies that both types of tests have been successfully passed, and the apparently irrational aspect has found a match with the rational one. However, in the opposite case, when one instead witnesses a clash between the AA and the RA, that is, the respective tests are not concordant, two different scenarios may emerge. In one case, the AA is the only one that is interested in the subject in question, deeming her/him a satisfactory partner according to its own evaluation criteria, whereas the RA showed no interest and thus failed to be excited and to create the TU. Alternatively, the TU has occurred but the AA finds the subject not acceptable as s/he failed to pass one or more filters that are relevant for the AA itself or for its socio-cultural context of reference.

## 5. Results: The Mating Stability Matrix (MSM)

When an opposition between AA and RA emerges, there is a conflict, meant as a proper internal struggle, be it personal or socialized, that prompts the individual to find a solution over time, with the eventual prevalence of an area over the other. However constraining and painfully persisting a TU, an extremely strong and affirmative AA, being the active part, may take over preventing the entrance into the TU-C or even forcing an exit from it. Thus, on the contrary, an accommodating AA may concede to the suffering of its own RA, which, despite being passive, will vehiculate a sense of unsustainability, even for the AA, of a life outside that very TU-C. In the former case, indirect rewards will be sacrificed, accepting the distressing, destabilizing consequences of what is commonly meant as a life that gives up to love. In the latter, instead, in which according to common sense love would be winning, what will be likely sacrificed are the direct rewards, maybe within the TU-C itself or externally, in the relational and/or socio-economic environment of reference—and this too is bound to have consequences on the TU-C as time passes.

The situation is further complicated if one considers that the TUs should be two, one for each partner, so that an optimal functioning of the TU-C becomes possible, and that at stake are the AA and RA of the opposite sex partner as well, which in turn may be opposed to one another. Conflict resolution, moreover, may require time, so that the TU-C may start its iterations with one or both participating partners into a state of inner conflict.

To orientate ourselves in the maze of possible options, simply articulating the concordance vs. opposition between the AAs and RAs of the partners of a couple that has formed, we will now introduce a matrix called the mating stability matrix (MSM, Table 1), which allows an assessment of the level of stability of a couple not only in terms of its position within the matrix itself, on the basis of the results from the compatibility and filter tests, and the presence or absence of a TU or of both, but also in terms of the possibility of movement in time within the matrix itself.

We then define the matrix’s labels by putting on the columns the possible options for M, and on the rows the options for F. We denote by (+) the success of a test (or of a group of tests, in the case of the filter tests) related to the area to which the plus sign is referred (AA or RA), and conversely by (**−**) the failure of that same test. In particular, in the case of the expression RA+, in addition to successfully passing the compatibility test for a given male or female subject, the (+) also denotes the emergence of the TU for M or F, depending on whether the (+) sign is found in the column vs. row labels. Likewise, in the expression RA**−**, the failure of the compatibility test and the consequent lack of a TU is indicated, whereas AA+ stands for an overall success of the filter tests and AA- for an overall failure of the filter tests.

Lucchi Basili and Sacco [12,13] show how the success of the compatibility test does not imply the mechanical emergence of the TU, and that often a number of iterations of the TU-C is needed so that the excitement of the RA grows to a level that crosses a minimal subjective threshold. In other words, it is necessary that indirect rewards, as generated by the RA, are gradually increased thanks to the TU-C until the reached level sparks the TU, which is subsequently fed and reinforced by any further iteration of the TU-C. To simplify these passages, in the matrix, the sign (+) referred to the RA denotes the whole process, from the success of the compatibility test to the emergence of the TU, even if the former normally does not necessarily imply the latter. However, as our matrix deals with already formed couples rather than with couples in the process of being formed, we presume that in the presence of a compatibility the TU has also eventually emerged. As to the (**−**) sign, again referring to the RA, the failure of the compatibility test in the matrix may indicate both the fact that the test has actually failed, with a current and future impossibility to lead to a TU, but also the possibility, however remote in an already formed couple, that the compatibility test has not been carried out yet, and that consequently the absence of the TU is not irrevocable in this specific case. This possibility has been contemplated to allow a less obvious type of movement within the matrix, in addition to classical ones due to the fragilization of the couple as a consequence of the dissolution of worn out TUs (something that may happen with time). For instance, a couple might have been forced into an arranged marriage set up by parents without giving the partners the possibility of a direct acquaintance, so that the couple finds itself in the bottom-right cell of the matrix (forced couple, see below). The movement within the matrix in this case might depend on the fact that we cannot rule out the possibility that, by living together, the initially mutually extraneous partners might enter into a well-functioning TU-C that brings about a TU or even a D-TU, consequently causing a movement toward different cells of the matrix.

In conclusion, by stacking the possible combinations of the states of each AA and of the respective RA, we will have four possibilities for each sex: Two concordant ones, with both areas taking a positive or negative sign, and two oppositional ones, where the two areas take contrary signs in turn. The matrix that results is therefore a 4x4 matrix with a total of 16 possible combinations between the AAs and RAs of the two subjects in their concordance vs. opposition.

Each cell in the matrix is identified by its (row, column) coordinates, where, in particular, for the F (row) subject, we denote, for instance, by F+**−** the row that corresponds to a concordance of the F-AA (+) and to an opposition of the F-RA (**−**) and likewise for M. Thus, the notation (F**−**+, M++) corresponds, for instance, to the cell at the crossing of the row (AA**−**, RA+) for F and of the column (AA+, RA+) for M.

The sector that consists of the first block of four cells in red, that is the cells (F++, M++), (F++, M**−**+), (F**−**+, M++), and (F**−**+, M**−**+), includes all mutually tied-up couples. The RAs are indeed all positive, with the launch of an operating TU-C, even if precarious in some cases. In particular, the cell that offers the best guarantees of stability is certainly the first, (F++, M++) because of the concordance between both AA-RA matchings. We will therefore call this sector the ‘tie-up sector’.

The sector that consists of the second block of cells, L-shaped in the matrix and in blue, includes the five combinations (F+**−**, M++), (F+**−**, M**−**+), (F+**−**, M+**−**), (F**−**+, M+**−**), and (F++, M+**−**), and is called the ‘exploitation sector’, where the term ‘exploitation’ indicates here the presence of a unilateral TU that gets exploited, or the total lack of TUs in a situation of reciprocal exploitation. In the exploitation sector, the TU-C is simulated by non-tied-up partners. The third and last sector is that consisting of the seven cells, the outermost L-form in the matrix, in brown. In this sector, the TU-C is entirely absent and the unilateral TU, when existing, is of a coercive type, in the sense that the tied-up subject affirms her/himself upon the non-tied-up partner, constraining him/her into the couple. This sector is called the ‘constriction sector’ and includes the combinations (F**−−**, M++), (F**−−**, M**−**+), (F**−−**, M+**−**), (F**−−**, M**−−**), (F+**−**, M**−−**), (F**−**+, M**−−**), and (F++, M**−−**).

### 5.1. The Tie-Up Sector

In the tie-up Sector, we find three variants of a TU within the couple, depending on the concordance vs. opposition of the AAs: The cooperative tie-up (F++, M++), the conflictual tie-up [(F**−**+, M++), (F++, M**−**+)], and the tragic tie-up (F**−**+, M**−**+). 

#### 5.1.1. Cooperative Tie-Up

Among all possible combinations, the one that presents a cooperative tie-up is the couple with better chances of long-term success, in that the concordance between all the areas creates the ideal conditions for super-cooperation between the partners and thus facilitates the optimal functioning of the TU-C, whose flow of rewards becomes self-propelling the more the cycle is iterated. The first top-left cell of the matrix then represents the point of higher stability in terms of TU-C. The term ‘cooperation’ is used here to emphasize the reciprocal collaboration between partners for the purpose of the optimal functioning of the TU-C, but also indicates the existence of a harmony that may lead to a commonality of interest or intent, that is, to a projectual couple of some kind. Nature has contemplated this kind of (super-)cooperative capability to experiment with joint creative strategies of child rearing, whose mode and content is appropriate and conformal to the intrinsic characteristics of each couple. However, this bio-behavioral programming does not rule out other, offspring-unrelated forms of projectual (super-) cooperation with other kinds of creative outcomes, possibly equally positive for the couple or for society. A couple that builds upon a shared, jointly pursued life project benefits from an extra source of stability and a better chance of resilience in time.

#### 5.1.2. Conflictual Tie-Up

A conflictual tie-up occurs when, despite the presence of a D-TU and of a functioning TU-C, an element of instability arises due to the opposition of one of the AAs, which generates a conflict in one of the partners, F or M. An example of conflictual tie-up is when one of the partners has to manage the consequences of a preexisting bond, such as children from a previous relationship, which is threatened by the formation of a new couple bond. More generally, the failure of a filter test of the AA that is pertinent to an aspect believed to be vital by the other partner may spark a conflict in the latter that, if unresolved, ends up pouring into the TU-C too many frustrations, which, by putting an obstacle to the flow of rewards, might cause the destabilization of the TU-C itself. The concordance between RA and AA is a factor that strengthens the TU, making it much stronger and safer with respect to the case where an internal conflict between areas in opposition arises. A TU that is only supported by the RA will therefore tend to dissolve more easily with time, and especially so if not properly fed by the flow of rewards of the TU-C.

In other cases, however, the conflict might even evolve to the benefit of the couple by building on the solidity of the common cooperative basis due to the presence of both TUs. An example is when the behavioral, cognitive, or affective limitations of a partner, although negatively assessed by the filter tests carried out by the AA of the other partner, may motivate the former to improve her/himself to accommodate the desires and the expectations of the latter, so as to be stimulated and supported by the other partner in her/his own path of personal development.

Of course, the possibility of conflict in a couple may arise for different reasons, and there may also be conflicts that derive from frictions in the functioning of the TU-C, unleashed, for instance, by emotional cues (insecurity, jealousy etc.) that are not prompted by the AA, by the failure of the filter tests, or by a lack of concordance between the AA and the RA. This further kind of conflict is not accounted for in the stability matrix.

#### 5.1.3. Tragic Tie-Up

The tragic tie-up occurs when the conflict due to the opposition of the AA concerns both partners at the same time. The tragic character derives from the impossibility of engaging in a vital TU-C despite the existence of ideal conditions. This is the case of the lovers that cannot see the fulfilment of their union due to the existence of tough obstacles that are hard to overcome, which block the formation or the maintenance of the couple. The already cited Shakespearian couple, Romeo and Juliet, provides a textbook example of a tragic tie-up. The TU in this story is literally tragic as it leads to the death of both young lovers. By analyzing the couple from the viewpoint of the filter tests, the one which fails is the social approval test, which in this specific case has a family-related nature. The lead characters in fact make no barrier of their own to their union. Even if the tests of familiarity and conformity are ruinously failed, the AAs of the young lovers manage nevertheless to preserve the concordance with their respective RAs. Such tests, instead, acquire a relevance that is external to the couple being formed, and become salient for the families, whose meddling determines the sign (-) found in the matrix.

The historical period and the country where the story takes place are crucial for the stability of the couple. Determining the failure of the familiarity test is the rivalry and tribal hatred between the two families of origin of the two characters, the Montecchi and the Capuleti, whose roots are too deep to be overcome with a marriage. Moreover, the conflict further escalates with the death of Mercuzio, the friend of Romeo killed by Tebaldo, the cousin of Juliet, and with the death of Tebaldo killed in turn by Romeo as a revenge. The consequent banishment of Romeo also sanctions the failure of the social conformity test.

### 5.2. The Exploitation Sector 

The sectors of the matrix that relate to exploitation and constriction include, among others, cases where the TU is unilateral, that is, in the couple we find only one tied-up partner. The stability matrix is built in such a way that the last two rows and columns correspond to negative values of the RA in the corresponding labels, so that when these rows and columns cross the first two rows and columns, we have a unilateral TU, which may be either susceptible to exploitation by the non-tied-up partner, or oppositely may provide the excuse for a subjugation, that is, a constriction of the non-tied-up partner. Which factor supersedes the materialization of one possibility over the other? If, for instance, we examine the last two cells of the first column, we see that in both M is tied-up, whereas F is not. What makes then of M a partner exploited by F in the next-to-last cell of the first column, and what makes instead of M a partner that abuses F in the last cell of the same column? The difference lies in the AA whose RA is not tied-up—in this example, the F-AA. If the latter has a (+) sign, that is, if the filter tests have been successful overall, this implies that the F-AA has found a personal interest to mate with M, even without the F-TU (F+**−**). It follows that, to tie M up and keep him involved, F will make use of the TU-C, simulating to be tied-up, that is, to have an intrinsic interest and drive toward the other that actually does not exist. If M gets tied-up, the TU-C is launched, but, because of the simulation of F, it will prove unstable. When F gets tired of pretending or loses her convenience, the TU-C will be in crisis and with it, most likely, the couple itself.

The simulation always has the purpose of inducing and exploiting the unilateral TU of the partner, to reap the benefits highlighted by one or more filter tests. In other words, exploitation is inherent in a simulated TU-C, because the D-TU is lacking, as one of the subjects is not really interested in rewarding the partner, not having any intrinsic interest toward her/him, whereas s/he is only interested in being rewarded her/himself.

If instead the AA of the non-tied-up F presents a negative sign (**−**), as in the last cell of the first column (F**−−**, M++), this means that the interest to build and maintain the couple will be only M’s, the one who is tied-up to F (M++). As F will tend to avoid M (F**−−**), the latter may strive to form the couple, this time without a functioning TU-C, only by enforcing a constriction of some kind upon F.

The last row and column of the matrix, being characterized by negative signs only in its labels, both row (F**−−**) and column (M**−−**), belong to the constriction sector, where the partner with at least a positive (+) sign, in the RA, the AA, or in both, proves to be the only one interested in the couple. Therefore, if the couple comes into existence, this means that the partner with the (+) sign has constricted the other, who would have fled otherwise. In the absence of (+) signs, that is on the main diagonal in the bottom-right cell of the matrix, where both partners are uninterested in the couple (F**−−**, M**−−**), the constriction inevitably becomes external to the couple itself.

Let us now examine in more detail the single cells of the exploitation sector, namely the five in blue, which contemplate two possibilities of exploitation: Exploitation of a remissive TU, and exploitation of a conflictual TU, for a total of four combinations, plus the opportunistic couple in the cell of the main diagonal of the matrix.

#### 5.2.1. Exploitation of a Remissive TU

The area that is interested in exploitation is always the AA with a positive sign (AA+), which accompanies a non-engaged RA (RA**−**), which remains indifferent, turned off, in other terms, non-excited. The reason of the lack of interest of the RA, from which it follows the absence of the TU, may depend upon the failure of the compatibility test, that is, the partner to whom one is mated is not biologically (M) or psychologically (F) compatible, or upon the fact that the compatibility test has not been carried out yet, so that the positioning in the matrix is temporary or, alternatively, not confirmed yet.

The TU-C is launched anyway, but it is not stable, as one of the partners is simulating to be tied-up, whereas the only existing TU within the couple is remissive, that is, accommodating. The accommodation is not really due to the fact that the partner is tied-up, but to the fact that there exists a concordance between her/his areas, that is, in addition to the RA, the AA is also positive (+), in that the filter tests have also been successfully passed. With the concordance of the areas, the involvement of the partner into the relationship is at its peak, and most likely the subject does not suspect any simulation on the side of the other, and thinks to sit in a different cell of the matrix, namely the top-left, that of the cooperative tie-up, and then, consequently, s/he unconditionally cooperates. The attitude that we have defined as remissive is in fact a unilateral cooperation. At times, finally, being remissive may be the outcome of a perverse combination of desire and insecurity that induces self-deceit, making the very possibility of the doubt that the other does not really have sincere feelings unacceptable.

#### 5.2.2. Exploitation of a Conflictual TU

This second case is analogous to the previous one, with the exception of the AA of the tied-up subject. This time, doubt about the sincerity of the involvement of the other may exist, and may even sink into obsessive suspect. The negative AA (**−**) when the RA is positive (+) might also mean that there are one or more failed salience tests, which might cause a lack of esteem or appreciation of a varying nature (according to the nature of the filter that has failed). The conflict that ensues will have an inner nature of opposition between areas: A highly excited RA and a contrarian AA, which despite disapproving of the choice does not succeed in revoking it by subjecting its own RA. The conflict may easily become apparent also within the couple, for instance, with crises of jealousy by a wife who has been repeatedly cheated with impunity, or by a husband who gets annoyed and feels threatened by the fact that his wife has rich personal interests and/or a successful professional life, as he perceives such activities as a subtraction of attention toward him. 

If in the above discussed case of remissive exploitation the damage to the TU-C and the couple breakdown depend on the resistance, in terms of duration, of a tiresome simulation or on the actual persistence of the benefits from remaining in the relationship, in the case of conflictual exploitation, the couple is even more at risk, as the conflict pours into the TU-C further frustration that displaces the rewards already drained by the selfishness and egocentrism of the exploiter. Moreover, it is reminded that in general the absence of the TU within the couple paves the way to the possibility that, in any moment, another TU external to the couple is created with an alternative potential partner—a situation that, more than any other, would destroy the TU-C.

#### 5.2.3. The Opportunistic Couple

We now are on the matrix’s main diagonal: This means that the labels are identical for the rows and columns, that is, in the case of this specific type of couple, both partners are not tied-up (RA**−**), but at the same time both have at least one reason to exploit the opposite sex partner (AA+). The opportunistic couple represents the peculiar but far from improbable case of a functioning TU-C where both partners simulate their interest toward the other, pretending that an actually non-existent D-TU is in place. Examples in this regard might be that of a loveless marriage, which, however, provides social benefits to both partners, in that it helps their social positioning, or also the achievement of a state of independence from parents, which is perhaps much longed for. In certain socio-cultural contexts, marrying someone may be so important that psycho-biological compatibility, or mere attraction of some kind, falls into the background, or even becomes irrelevant. In this case, the opposition between RA and AA evolves into a predominance of the needs and of the tests of salience of the AA.

### 5.3. The Constriction Sector

The outermost part of the matrix, occupied by the constriction sector, consists of seven cells in brown. This peripheral sector is the one that may more easily host situations of abuse, violence, or psycho-behavioral deviance, in addition to a plain and simple propensity to bullying. In this sector, the TU-C is never formed, and at any rate it could not survive due to the lack of a circulation of rewards inside the couple. The constriction of a partner may have a solipsistic, appropriative, or opportunist nature, and may apply to both sexes, for a total of six combinations. Moreover, in the last cell on the main diagonal, we find the forced couple. The subject that suffers from the constriction has minus signs (**−**) in all areas, so that there is a concordance between AA and RA, but it is negative. Therefore, no compatibility with the partner has been found, there is no TU, and there is not enough relevance in terms of filter tests that may make the union with the partner rewarding in any sense. Yet, the latter has found a way to block the recalcitrant partner within the couple, possibly even by means of extortion.

#### 5.3.1. Solipsistic Constriction

The subject who operates a solipsistic constriction presents a positive concordance, whereas the subject that suffers it has a negative concordance [(M++, F**−−**), (F++, M**−−**)]. This terminology serves to underline how a partner may selfishly consider her/his own viewpoint as the only reason to request the formation of a couple where the other’s intentions and will simply do not matter. In certain cultures or epochs, especially when a hierarchical patriarchal structure prevails, the fact that the will of only one partner may have weight, relevance, and meaning is socially legitimized and thus undisputed, and often also defended through gender discrimination. However, it may also happen that a single individual, not necessarily male, simply follows her/his caprice, and has the opportunity to perpetrate an abuse, by exerting some personal position of power on the other. This is a case where the TU has disqualifying implications for the tied-up subject, and where the altruistic emphatic component, which normally tends to be amplified with the TU, here does not develop at all. Think, for instance, of a subject that displays ego-centric attitudes and an extreme subjectivism, and who, once tied-up, literally demands reciprocation. Such a pretense appears to the subject as natural and obvious, as if being loved is something due to the one who loves, or thinks to love, so intensely.

#### 5.3.2. Appropriative Constriction

For appropriative constriction, the same remarks made for solipsistic constriction still hold, with only one difference: The AA of the abusive subject is negative (AA**−**), that is, s/he does not deem the subject s/he is tied-up to worthy of her/him, for some reason despising him/her or considering him/her as inferior [(M**−**+, F**−−**), (F**−**+, M**−−**)]. The subject who is constricted into the couple relationship is instrumentalized, as if being an object which can be appropriated, a commodity that is literally purchased, becoming a possession to be used for one’s own necessities, without any concern for the other. This type of couple shows unquestionably how dominance, not permitting an egalitarian manifestation of thoughts, sentiments, and personalities, makes the functioning of any TU-C simply impossible. Typical, in this sense, is the couple where the tied-up subject develops an obsessive fixation for the partner, localized in the RA, which is, however, accompanied by elements of devaluation and contempt, due to the negative orientation of the AA. In particular, such an obsessive fixation will assume a sexual dimension if the tied-up partner is female, with a devaluation of the partner’s capacities that may be the object of social appreciation, and to the contrary a fixation on attention/recognition if the tied-up partner is male, with a female devaluation mainly focused on her sexuality, desirability, and integrity. An example is that of the man that diminishes the partner by unfavorably comparing her to goods with a strong valence of identity recognition, and thus with a sexual implication in the case of the M-AA, such as, for instance, a luxury car that may more effectively and satisfactorily sanction the owner’s masculinity.

Moreover, we can observe that the diminished partner receives repeated frustration, which mainly aims at her/his RA (being the opposite sex partner AA the one that drives the attack), so that the absence of the TU, in the partner that presents a negative concordance, is consequently inevitable.

#### 5.3.3. Opportunistic Constriction

The last form of constriction on a subject with a negative concordance is the opportunistic one, which characterizes an AA+ in the absence of a TU ((F+**−**, M**−−**), (M+**−**, F**−−**)). There is no bond on either side, but one of the partners has some personal advantage from being in the couple. Within the matrix, the cell of the opportunistic constriction sits in the row or column of exploitation (AA+, RA**−**), so that we can regard opportunism as a form of exploitation that in this case is declared and not concealed, that is, there is not even the need to pretend to be tied-up. This is because pretending the existence of a bond would not generate any additional benefit given that the other is in turn not tied-up and has no interest in being so. An example in this regard is a marriage of convenience, set up by the families to the benefit of the families themselves, reflecting the will of a single partner.

#### 5.3.4. Forced Couple

The antithesis of a couple with a cooperative tie-up is the forced couple, at the opposite side of the matrix’s main diagonal, with a negative concordance of all areas (F**−−**, M**−−**). The couple is forced because none of the partners have any kind of interest in the other, so that the couple could not spontaneously exist. Clearly, the forcing is exerted outside the couple, and is determined by circumstances. A typical example is that of the marriage of reparation, when honor and thus social conformity, not only of a single subject but of the entire family of origin, is put at stake by the birth of an out-of-wedlock child, lacking the legitimization of the community. 

The forced couple provides an extreme demonstration of the concrete possibility of a mating independently of the presence of a functioning TU-C, and of how the formation of the couple needs salience and ties of some nature, be them spontaneous, simulated, or imposed. However, its actual durability requires that, if the couple is based upon instrumental benefits or constriction, such convenience or constriction may effectively persist in time. The alternative possibility is the TU-C, where a well-fed flow of rewards can function as a real engine of psycho-relational and affective growth for the couple in the absence of any need of external constraint.

### 5.4. Stability within the Matrix and the Adaptive Value of the TU-C

The stability of the couple as a function of its positioning within the MSM is variable, depending on the socio-cultural contexts and the historical periods. This is because, despite the stability provided by a well-functioning TU-C is undeniable, socio-economic pressures may over time condition it from the outside, in a more or less pressing and meddling way, pouring in a steady amount of frustration into the cycle. For this reason, if ideally the tie-up sector represents the most stable area, with better guarantees of durability, it is also true that this sector necessitates the existence of conditions of parity between the partners, both as regards an equal recognition of gender dignity and as to the rights and responsibility of both partners toward the couple, so that the TUs may effectively unleash their entire potential in terms of cohesive force and resilience to difficulty. 

The more severe the inequality between genders, together with a cultural and socio-political structural rigidity that preserves the incumbent social order while inhibiting the freedom of choice of the single individuals, the greater the relevance of the exploitation and constriction sectors in guaranteeing the duration and stability of the couple. This would explain, for instance, in a context of strong dominance as deeply rooted in patriarchal societies, the fragility of the so-called love marriage, with respect to the safer marriage of convenience, supported by a cohesive force that is external to the couple rather than internal. The latter remark stresses the difficulty of drawing general inferences as to the comparative inclusive fitness of various types of couples irrespectively of the specific social environment in which they are embedded. In particular, it cannot be said in principle that couples where one or both partners are tied-up are necessarily more adaptive to a given environment than couples where tie-ups are not found. We have shown that the tie-up cycle has an evolutionary rationale that suggests that reciprocally tied-up couples may be adaptive in some circumstances due to their cohesiveness and dynamic resilience to external shocks. However, characterizing such circumstances with some precision is still an open question. Therefore, investigating how the characteristics of different social environments impact upon the fitness of the various types of couples in the MSM is an important and fascinating topic for future research, with significant implications for cross-cultural psychological inquiry. 

The concordance vs. opposition between the areas of a single subject, both in the presence of a TU and in its absence, provide important indications as to how the subject will tackle the interaction with a potential partner. The formation of the couple may entirely do without the existence of the TU-C, but the presence of a well-functioning, non-simulated TU-C allows the possible development of a fusional (super-) cooperative mode among partners that self-feeds through interaction, and is not achievable otherwise. When the conditions for the functioning of the TU-C persist over time, by analyzing the concordance vs. opposition between the areas of the two partners, it will become possible to prefigure to some extent the unfolding, pace, and intensity of the iteration as a function of how much the subjects themselves contribute to feed it or, on the contrary, to obstruct it. 

As it is not possible to reduce the success chances of a couple to the mere outcome of the filter and compatibility tests without also taking into account whether both TUs have actually been created, likewise the TUs are not in turn sufficient without a good functioning of the TU-C, or worse, in its absence. If, therefore, the RAs may effectively impose themselves upon the frictions, by exploiting the drive of their excitement, a good functioning of the TU-C remains the indispensable condition for the stability of the couple irrespectively of the resilience of the TUs and, even less, of the success of the tests. This is because it is not possible to consider the success of a couple in a non-dynamic sense. Coping with the disruptive effects of the everyday routine, changes, evolutions, and perturbations of the life course calls for an equally dynamic system that allows, from time to time, a constant self-repurposing that does not rule the partner out in the joint pursuit of a healthy and vibrant couple bond. 

The TU-C represents a system that is not only dynamic, and thus capable of flexibly adapting to mutations, but especially, once launched, geared toward self-reinforcement: A fusional couple is not worn out by interaction, but on the contrary is regenerated and maintains its stability over time, with a clear adaptive valence. The TU-C with its iterations works as a sort of flywheel: It gathers energy and dampens the variations in speed (of the flow of rewards) that occur when one of the partners caves in. This is the reason of the support that the couple, as an operating relational cycle, is able to offer to the single partners. Analogously, the TU-C, as a flywheel, is responsible for the impulse to the human development of each partner.

## 6. Discussion and Conclusions

Although the MSM contemplates a wide variety of possible types of couples from the point of view of the modulations of concordance/opposition between one’s own AAs and RAs and the partner’s ones, none of these possibilities represents a mere theoretical speculation. There are strongly cooperative couples that arrive at a true fusion of personalities and life projects, as well as couples marked by abuse and by physical and psychological violence. There are couples where the adhesion of the partners is intentional and founded on an intrinsic motivation, even if possibly marked by the internal conflicts of one of the partners or of both, but there are also couples where such adhesion is dictated by opportunistic motives or by a real constriction of one of the partners and even of both. None of the possibilities featured in the MSM are too extreme to fail to be populated by a large number of real cases. 

Human couples present an extraordinary variety of modes of relation, and a theory that aims at describing and interpreting the processes behind their formation and functioning should be able to envision and possibly explain such variety. The tie-up theory proposes a conceptual framework that stresses certain factors as being responsible for this variability, and emphasizes how the presence or absence of the TU in one or both partners represents a discriminating element for the level of cooperation within the couple and for its stability against the changes in external circumstances. On the other hand, the MSM shows how only a limited number of couple types are really founded on a double tie-up, which therefore cannot be considered as a standard of reference in the analysis of real couples. The onset of the D-TU is possible only in particular conditions, which probably describe only a minority of actual human couples. Yet, the couple characterized by a D-TU has a particular cultural salience in the human romantic imaginary [13], because it represents an adaptive solution of special value for the partners who manage to build it, in that it realizes an endogenous correspondence, which is the fruit of a common super-cooperative investment, between two subjects whose reciprocally ideal character is not predetermined, but co-created.

In light of the taxonomy of possible couples as defined by the MSM, approaches to couple formation, such as Becker’s marriage market, present the clear limit of focusing on a particular type of couple, in this specific case the opportunistic couple, making it a standard of reference in its being modelled by the configuration of incentives that is believed to be most ‘realistic’ for the two partners. What the tie-up theory shows is that the function of the TU-C is exactly that of causing a gradual cooperative focusing of the two partners, so as to prevent the couple being structured as a temporary association driven by opportunistic motives. The couple instead becomes a cooperative unit founded on deep mutual trust and on a cohesion that is not conditioned upon the partners’ capacity to provide a flow of rewards that is larger than what could be obtained from possible alternative partners.

It is on the basis of these premises that the couple manages to exist in a medium- to long-run horizon even in the absence of external constrictions. In a couple where the possibility of an opportunistic defection by one of the partners as circumstances change would become common knowledge, many of the most functional and adaptive aspects of the couple would vanish in view of the partners’ constant concern of protecting themselves from the consequences of a possible defection of the other, with the consequent dissolution of any real investment in the construction of an affective intimacy, of mutual trust, and so on. The opportunistic couple compensates this gap by turning marriage into a contract, which explicitly translates the choice to remain in the couple into the result of a cost-benefit evaluation.

As already observed, the couples that are based on a D-TU are likely a minority, but they do not represent some deviant behavior with respect to a presumed opportunistic ‘norm’. The process of couple formation, in ‘normal’ conditions, is not the result of a rational deliberation, but of a complex interaction between conscious and sub-conscious elements, which may now be finally characterized and understood in neuro-scientific terms. The aspect of the tie-up theory that may have interesting implications for a future cycle of experimental research is explaining how the process of couple formation may be driven by forces that are different from those that the partners themselves tend to identify through ex ante or ex-post rationalizations of their choices. Interrogating human subjects as to their preferences and the desirable characteristics of a possible partner means to probe a specific dimension, that of the active areas that are conscious and sensitive to the action of social forces. It is in the receptive areas, however, and not in the active ones, that the action of the biological, psychological, and behavioral programming that is at the root of the intrinsic motivation of the two partners to form a long-term couple is fully deployed.

The very notions of short- and long-term couples conceal in fact a variety of situations that are difficult to categorize by means of such a generic terminology. There may be long-term couples characterized by situations of substantial exploitation or abuse of a partner over the other, which are kept stable by a certain socio-cultural context, and there may also be short-term couples whose cause of dissolution is not of an opportunistic nature but, to the contrary, is altruistic, such as in the case of the operation of the moral responsibility filter that leads a tied-up partner to leave the couple to altruistically prevent the other partner from suffering a major damage. More than the couple durability, the conceptually distinctive element to structure a taxonomy of couples seem to be that of the existence (or of the failed existence) of the TU, and its possible unilateral or bilateral nature.

It is clear that to test its descriptive and predictive validity, this approach needs to go through an extensive experimental check. A first research path of some interest is that of the analysis of narrative corpora characterized by a certain level of social validation, which thanks to the social cognition valence of fictional narratives [14] may provide a first test bed to check to what extent the structure of socially validated romantic narratives tends to reflect the implications of the tie-up theory. Early analyses carried out on some of the most successful Hollywood romantic comedies of the last decades [13] and on the main romantic fairy tales [46] offer a first, albeit preliminary, corroboration of the theory, thus encouraging further research on the topic. It is now necessary to develop an experimental strategy as well, which takes into account the caveats already brought out in the previous discussion, namely avoiding the elicitation of the preferences of the subjects in an abstract context and in a plainly artificial situation. A wave of experimental studies that are robust against this type of criticism are therefore called for, and this is certainly a challenging task for future research. Some alternative paths may be those of the semantic analysis of individual accounts of real within-couple experiences, or, in a complementary rather than alternative logic, the measurement of the psycho-neuro-endocrinological response to certain stimuli targeting the conscious and sub-conscious reward systems of experimental subjects, so as to highlight possible distinctive signatures of the activity of the AAs and RAs. This would help to overcome the limits of an elicitation based upon self-perception and self-representation, to directly probe into the circuits of the bio-behavioral programming of mating choices.

In light of the big research effort put in the last decades into the topics of the formation and stability of human heterosexual couples, we believe it is important to stress the necessity of a theory that does not conceptualize the choices related to couple formation in terms of abstract categories of desirability or of artificially restricted sets of motivations. Instead, there is a need to develop increasingly sophisticated tools to understand how the combination of the characteristics, expectations, and desires of two specific potential partners, and their direct interaction, may be conducive or not to the formation and stability of a more or less cooperative, fusional, and accomplished couple, as opposed to opportunistic, or based upon exploitation, or upon constriction. Only time will tell what extent the experimental evidence will support these theoretical intuitions.

## Figures and Tables

**Table 1:** The mating stability matrix (MSM).

	M	AA+ RA+	AA− RA+	AA+ RA−	AA− RA−
F					
AA**+** RA**+**		Cooperative TU	Conflictual M-TU	Exploitation of Remissive F-TU	Solipsistic Constriction by F
AA**−** RA**+**		Conflictual F-TU	Tragic TU	Exploitation of Conflictual F-TU	Appropriative Constriction by F
AA**+** RA**−**		Exploitation of Remissive M-TU	Exploitation of Conflictual M-TU	Opportunist Couple	Opportunistic Constriction by F
AA**−** RA**−**		Solipsistic Constriction by M	Appropriative Constriction by M	Opportunistic Constriction by M	Forced Couple

Legend: + successful test; **−** failed test; AA+ overall success of the filter tests; AA**−** overall failure of the filter tests; RA+ success of the Compatibility Test and TU; RA**−** failure of the Compatibility Test and lack of TU. Colors Legend: Tie-Up Sector (red) ==> well-functioning TU-C ==> RA**+** ==> D-TU. Exploitation Sector (blue) ==> simulated TU-C ==> exploited unilateral TU or absence of TU. Constriction Sector (brown) ==> absent TU-C ==> coercive unilateral TU or absence of TU.
